# Performance of quantile regression methods with discrete outcomes: A simulation study with applications to environmental epidemiology

**DOI:** 10.1097/EE9.0000000000000432

**Published:** 2025-10-28

**Authors:** Joshua D. Alampi, Bruce P. Lanphear, Lawrence C. McCandless

**Affiliations:** a Faculty of Health Sciences, Simon Fraser University, Burnaby, British Columbia, Canada

**Keywords:** Bayesian statistics, Discrete data, Quantile regression, Simulation study

## Abstract

**Background:**

Quantile regression helps identify how associations vary across the outcome variable’s distribution. Using simulations and data from the Maternal-Infant Research on Environmental Chemicals study, we showed that frequentist quantile regression can produce implausible results where the point estimates are integers or rational numbers and the outcome variable is discrete, which is common in health research. Applying “dithering” (also known as jittering) or using Bayesian quantile regression can prevent such implausible results, but the optimal strategy is unclear.

**Methods:**

We conducted simulations with discrete outcomes to compare the bias and variability of point estimates of undithered frequentist, dithered frequentist, and Bayesian quantile regression. We also compared the coverage and interval-width variance of these methods’ confidence or credible intervals.

**Results:**

The dithered frequentist method generated point estimates that were less variable than the undithered frequentist method. The Bayesian method had the least variable point estimates, but when the sample size was low (n = 100), it exhibited bias when modeling a binary or discrete covariate. The dithered frequentist method with xy-bootstrapped confidence intervals had nominal coverage and produced intervals with relatively consistent widths. The Bayesian method with adjusted intervals also had nominal coverage, but more variable interval widths. The Bayesian method with unadjusted intervals had poor coverage.

**Conclusion:**

In our simulations with discrete outcomes, dithered frequentist quantile regression (particularly with xy-bootstrapped confidence intervals) had the best overall performance. The Bayesian method with adjusted intervals is an acceptable strategy, although it was biased under certain scenarios and generated credible intervals with more variable widths.

**What this study adds**

Quantile regression models the exposure-outcome associations throughout the outcome variable’s distribution. Using data from the Maternal-Infant Research on Environmental Chemicals Study and simulations, we showed that frequentist quantile regression can generate implausible results with discrete outcomes. “Dithering” or using Bayesian quantile regression can prevent such implausible results. We conducted simulations to compare the performance of undithered frequentist, dithered frequentist, and Bayesian Quantile regression. Our results suggest that dithered frequentist quantile regression has better performance than undithered frequentist quantile regression when the outcome variable is discrete. Bayesian quantile regression is an acceptable alternative, although it had mixed performance in our simulations.

## Introduction

Epidemiologists frequently rely on ordinary least squares (OLS) regression techniques (e.g., linear, logistic, and Poisson regression) to estimate the average effect a given risk factor has on an outcome. These techniques assume that the entire outcome distribution “shifts” uniformly in response to a risk factor. The “shifts in the mean” that OLS regression techniques identify can have important public health implications.^1,2^ In particular, a small shift in the mean of the distribution may substantially increase the number of people in the tails of the distribution who cross a threshold associated with poor outcomes.^1–4^ In addition to considering the shifts in the mean, one can complement their OLS analysis by looking beyond the mean with quantile regression (QR). QR is an easy-to-implement statistical method that estimates the associations between risk factors and outcomes at a specified quantile (denoted by τ) of the outcome variables’ distribution.^5^

QR can determine if a risk factor is more strongly associated at the upper or lower tail of the outcome distribution, which can uncover important nuances.^6^ For example, while the average body mass indices (BMIs) in Mississippi adults has increased over time, this increase has largely been driven by the BMI distribution becoming more right-skewed.^7^ This suggests that we need to intervene at the upper tail, not just the mean. Next, a paper that used QR found that environmental and social stressors (lead exposure and low socioeconomic status) were most strongly associated with decreased test scores among lower performing children.^8^ Average associations (using linear regression) were weaker.^8^ This suggests that the children with the lowest test scores (who may be of particular interest) are particularly susceptible to environmental and social stressors. This also suggests that these exposures did not simply shift the distribution of test scores uniformly, but “stretched” the distribution toward the left tail.^8^ Despite the potential utility of QR, relatively few studies in environmental epidemiology or health research in general have used it.^8–25^

### Hurdles in implementing QR with discrete outcomes

Despite the potential benefits of QR, uncertainties on how to best implement QR may discourage epidemiologist from using it. Specifically, QR relies on the outcome variable having a smooth and continuous distribution for proper inferences.^26^ When the outcome is not smooth and continuous (i.e., the outcome is discrete), QR can produce an implausible pattern of point estimates ($\beta_{\tau }$). We show this using data from the Maternal-Infant Research on Environmental Chemicals (MIREC) study (further described in section The MIREC study). We modeled the adjusted associations between gestational metal concentrations and childhood Social Responsiveness Scale (SRS) scores (indicative of autistic-like behaviors). Crucially, SRS scores are discrete, they are whole numbers that lack a smooth and continuous distribution. When metal concentrations were a binary variable (modeling having > or ≤ median concentrations), all QR point estimates were integers (i.e., $\beta_{\tau }$ = −2, −1, 0, 1,...) or rational numbers (i.e., fractions; $\beta_{\tau }=-\frac{4}{3},\;-1,\;-\frac{2}{3},-\frac{1}{2},\;-\frac{1}{3},\;0,\;\frac{1}{4},\;\frac{1}{3},\;\frac{1}{2},\;1$) (Table 1, Table S1; https://links.lww.com/EE/A382). When chemical concentrations were continuous (specifically they were log_2_-transformed to achieve an approximately normal distribution), some point estimates were *exactly* zero ($\beta_{\tau }$ = 0) (see Cadmium and Mercury when τ=0.5;  Table 1, Table S1; https://links.lww.com/EE/A382), and others were real numbers. We emphasize that it is implausible that the true parameter would be equal to an exact integer (i.e., $\beta_{\tau }$ = 0) or fraction (i.e., $\beta_{\tau }$ = $\frac{1}{2}$), so it is concerning that frequentist QR may be limited to reporting such point estimates when outcomes are discrete. Additionally, several instances existed where frequentist QR’s rank-based confidence intervals were asymmetric (see Cadmium; Table 1, Table S1, Figure S1; https://links.lww.com/EE/A382).

**Table 1.** Point estimates (and 95% intervals) for the associations^a^ between first-trimester metal concentrations and child SRS scores (indicates autistic-like behaviors) using various quantile regression methods, the MIREC Study, Canada, 2008–2011 (n = 568)

Quantile regression method	Chemical name	$\beta_{\tau }$ (95% intervals)		
		τ = 0.1	τ = 0.5	τ = 0.9
Binary chemical concentrations (above versus below median)				
Standard (undithered) frequentist^b^	Arsenic	−0.5000 (−0.9560, 1.2897)	0.2500 (−1.0193, 1.5129)	−0.6667 (−2.5084, 0.9878)
	Cadmium	−1.0000 (−1.0000, 0.6646)	1.0000 (−0.6215, 1.0000)	1.0000 (−0.8748, 3.7520)
	Lead	0.5000 (−0.9416, 1.4345)	0.0000 (−1.1454, 1.5533)	0.3333 (−1.4761, 1.9343)
	Mercury	−0.6667 (−1.4153, 0.7582)	−0.3333 (−1.7160, 0.9206)	−1.3333 (−3.4121, 0.1234)
Dithered frequentist^b^	Arsenic	−0.3162 (−1.1462, 1.1535)	0.4962 (−1.0361, 1.2205)	−0.6729 (−2.4828, 1.2481)
	Cadmium	−0.1566 (−1.1320, 0.8887)	0.7034 (−0.6741, 1.6000)	0.8932 (−0.9564, 3.6444)
	Lead	−0.1327 (−0.9530, 1.3664)	0.1195 (−0.9775, 1.4388)	0.0416 (−1.7129, 1.7562)
	Mercury	−0.5095 (−1.3300, 0.4815)	−0.3711 (−1.4653, 0.8925)	−1.4129 (−2.8419, −0.1503)
Undithered Bayesian^c^	Arsenic	−0.1238 (−0.9248, 0.6601)	0.2804 (−0.3013, 0.8637)	−0.7753 (−1.7708, 0.2731)
	Cadmium	−0.2732 (−1.0250, 0.4715)	0.5297 (−0.0790, 1.1193)	1.2340 (0.1367, 2.3453)
	Lead	0.3505 (−0.4341, 1.1133)	0.1584 (−0.4905, 0.8259)	0.2538 (−0.7325, 1.2767)
	Mercury	−0.2809 (−1.0728, 0.5215)	−0.3149 (−0.9581, 0.3165)	−1.3923 (−2.3334, −0.3920)
Continuous log2-transformed chemical concentrations				
Standard (undithered) frequentist^b^	Arsenic	−0.1990 (−0.8641, 0.6308)	−0.0420 (−0.7908, 0.3053)	0.3153 (−0.8368, 1.3799)
	Cadmium	−0.4042 (−0.8650, 0.2812)	0.0000 (−0.5218, 0.7291)	0.4162 (−0.6869, 1.7989)
	Lead	0.5169 (−0.6441, 1.2583)	0.2014 (−0.6603, 1.3104)	0.4057 (−1.5029, 1.9234)
	Mercury	−0.2146 (−0.6073, 0.2022)	0.0000 (−0.4614, 0.2788)	−0.1916 (−0.7945, 0.4429)
Dithered frequentist^b^	Arsenic	−0.2295 (−0.9649, 0.5631)	−0.1194 (−0.7562, 0.4303)	0.1535 (−0.9151, 1.6073)
	Cadmium	−0.4292 (−0.7748, 0.2452)	−0.0145 (−0.4159, 0.7709)	0.3559 (−0.6320, 1.9285)
	Lead	0.5043 (−0.5687, 1.0906)	0.2482 (−0.6724, 1.2712)	0.2076 (−1.1843, 2.0486)
	Mercury	−0.1761 (−0.5767, 0.1180)	−0.0108 (−0.4565, 0.2291)	−0.3408 (−0.9080, 0.5037)
Undithered Bayesian^c^	Arsenic	−0.2238 (−0.6999, 0.2455)	−0.1629 (−0.4933, 0.1501)	0.2065 (−0.3772, 0.7908)
	Cadmium	−0.3964 (−0.7976, 0.0075)	0.0222 (−0.2904, 0.3427)	0.5015 (−0.0486, 1.0431)
	Lead	0.5083 (−0.0438, 1.0527)	0.2982 (−0.2225, 0.8085)	0.7461 (−0.0608, 1.5330)
	Mercury	−0.1801 (−0.4738, 0.1095)	−0.0378 (−0.2382, 0.1539)	−0.2390 (−0.5739, 0.1001)

a Controls for child sex, family income, maternal education, maternal age, self-identified maternal race, parity, maternal cigarette smoking during pregnancy, and whether the mother lives with their partner. Point estimates are rounded to four decimal places to demonstrate that many of them are rational numbers or integers.

b The default rank-based confidence intervals are used. See Table S1 or Figure S1; https://links.lww.com/EE/A382 for results with xy-bootstrapped confidence intervals.

c The default unadjusted posterior credible intervals are used. See Table S1 or Figure S1; https://links.lww.com/EE/A382 for results with adjusted posterior credible intervals.

MIREC indicates Maternal-Infant Research on Environmental Chemicals Study; SRS, Social Responsiveness scale.

**Figure 1.**
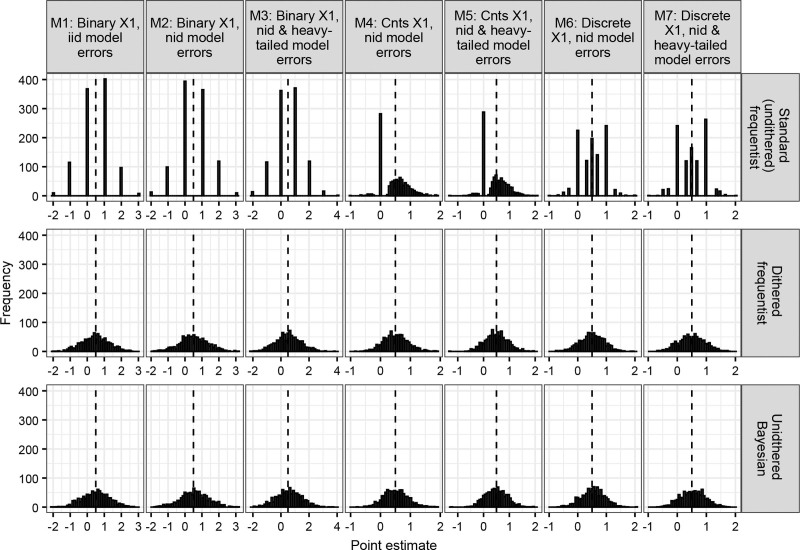
Histogram of point estimates from quantile regression methods in simulated data when n = 250 and τ = 0.5. The vertical dashed lines denote the true effect of $x_{1}$ on y. See Figure S2; https://links.lww.com/EE/A382 for other sample sizes and τ s. iid indicates independent and identically distributed errors; nid, nonindependently distributed errors.

These implausible results are more clearly demonstrated with simulated data with a discrete outcome variable (described in the section Data-generating mechanisms). Figure 1 (top row) depicts a histogram of point estimates from QR applied to simulated data with n = 250 datapoints and when modeling the median of the outcome (τ = 0.5). When a binary covariate ($x_{1}$) is modeled (models 1–3), all point estimates are integers ($\beta_{\tau }$ = −2, −1, 0, 1, 2, …). When a continuous covariate ($x_{1}$) is modeled (models 4 and 5), many point estimates were equal to exactly zero (Figure 1). When $x_{1}$ was discrete (i.e., taking on values of 0, 1, 2, or 3, simulating a “quantized” exposure variable where a one-unit increase denotes a one-quartile increase in exposure, models 6 and 7), all point estimates were integers or rational numbers ($\beta_{\tau }$ = −1, $-\frac{1}{3},$ 0, $\frac{1}{3},\;\frac{1}{2},$ $\frac{2}{3},$ 1, …). This confirms what was observed in the MIREC study. Consistent trends were observed for all other sample size and τ combinations (Figure S2; https://links.lww.com/EE/A382).

These implausible results (i.e., integer or fraction point estimates) are because of how frequentist QR calculates point estimates ($\beta_{\tau }$). It uses the simplex algorithm for model fitting wherein $\beta_{\tau }$ must be within the set of all slopes between the covariates and the outcome variable.^5^ When the outcome variable is discrete, then the distribution of possible $\beta_{\tau }$ values may be discrete. This resembles how the median of a set of numbers is within that set (assuming an odd number of values).

### Alternative strategies for QR with discrete outcomes

Alternative strategies for conducting QR with discrete outcomes include using dithered (also known as “jittered”) frequentist QR^26^ and Bayesian QR.^27^ Dithering adds uniformly distributed random noise to the outcome variable, giving it an artificially smooth and continuous distribution necessary for frequentist QR.^26^ Bayesian QR uses a Metropolis-Hastings algorithm to generate a posterior distribution of Markov Chain Monte Carlo (MCMC) draws.^28^ The mean of this distribution is the point estimate. Thus, Bayesian QR does not use the simplex algorithm, and it does not require the outcome variable to have a smooth distribution. When dithered frequentist QR and Bayesian QR were applied to the MIREC example, no implausible results were observed; all point estimates were real numbers, not integers or rational numbers (Table 1, Table S1, Figure S1; https://links.lww.com/EE/A382). Furthermore, our simulations on a discrete outcome show that both methods always generated a smooth, approximately normal distribution of point estimates, as expected (Figure 1, Figure S2; https://links.lww.com/EE/A382).

“Standard” (undithered) frequentist QR’s tendency to produce implausible point estimates of exact integers or fractions when the outcome variable is discrete could distort the results. This suggests that undithered frequentist QR is a poor estimator of the true parameter value when the outcome variable is discrete. This is a particular concern for children’s health research because many of the performance scales used to measure child development (e.g., SRS scores,^29^ intelligence quotient (IQ) scores,^30^ and anatomical measurements rounded to the nearest unit) are discrete. To our knowledge, however, no study has comprehensively examined the finite sample performance of undithered frequentist QR versus dithered frequentist QR or Bayesian QR on discrete outcomes.

In this paper, we will compare the performance of “standard” (undithered) frequentist QR, dithered frequentist QR, and undithered Bayesian QR on simulated data with a discrete outcome variable.

## Methods

We compared the performance of QR methods with a discrete outcome variable at the quantiles τ = {0.1, 0.5, 0.9} with a Monte Carlo simulation study that was repeated N = 1,000 times. All analyses were performed in R version 4.5.1. The frequentist and Bayesian approaches utilized the quantreg^31^ and BayesQR^28^ software packages, respectively.

### Data-generating mechanisms

We considered several models for generating simulated data that approximate an environmental epidemiology study. We anticipate that they will apply to a wide variety of scenarios both within and outside of health research. These models are as follows:

$$\begin{array}{lll} y_{i}= & \beta_{0}+\beta_{1}x_{1_{i}}+\beta_{2}x_{2_{i}}+\alpha_{y}\epsilon_{i};x_{1}\; & ∼Bernoulli\left(0.48+\;\gamma x_{2_{i}}\right);\;\epsilon ∼N\left(0,1\right) \end{array}$$ (1)

$$\begin{array}{lll} y_{i}= & \beta_{0}+\beta_{1}x_{1_{i}}+\beta_{2}x_{2_{i}}+\left(\alpha_{y}+\alpha_{x_{1}}x_{1_{i}}\right)\epsilon_{i};x_{1}\; & ∼Bernoulli\left(0.48+\;\gamma x_{2_{i}}\right);\;\epsilon ∼N\left(0,1\right) \end{array}$$ (2)

$$\begin{array}{lll} y_{i}= & \beta_{0}+\beta_{1}x_{1_{i}}+\beta_{2}x_{2_{i}}+\left(\alpha_{y}+\alpha_{x_{1}}x_{1_{i}}\right)\epsilon_{i};x_{1}\; & ∼Bernoulli\left(0.48+\;\gamma x_{2_{i}}\right);\;\epsilon ∼t\left(4\right) \end{array}$$ (3)

$$\begin{array}{lll} y_{i}= & \beta_{0}+\beta_{1}x_{1_{i}}+\beta_{2}x_{2_{i}}+\left(\alpha_{y}+\alpha_{x_{1}}x_{1_{i}}\right)\epsilon_{i};\; & x_{1}∼N\left(\gamma x_{2_{i}},1\right);\;\epsilon ∼N\left(0,\;1\right) \end{array}$$ (4)

$$\begin{array}{lll} y_{i}= & \beta_{0}+\beta_{1}x_{1_{i}}+\beta_{2}x_{2_{i}}+\left(\alpha_{y}+\alpha_{x_{1}}x_{1_{i}}\right)\epsilon_{i};\; & x_{1}∼N\left(\gamma x_{2_{i}},1\right);\;\epsilon ∼t\left(4\right) \end{array}$$ (5)

The errors (represented by ϵ) are homoscedastic (independently and identically distributed) in model 1, and are heteroscedastic (nonindependently distributed) in models 2–5 (as the inclusion of the quantity $\alpha_{x_{1}}$ in the model results in the variance of errors being dependent on $x_{1}$). The quantity $x_{1}$ follows a Bernoulli distribution in models 1–3, simulating a binary exposure variable that equals one 50% of the time after accounting for $x_{2}$. The quantity $x_{1}$ follows a normal distribution in models 4 and 5. Errors are normally distributed in models 1, 2, and 4. Errors have a *t*-distribution with 4 degrees of freedom in models 3 and 5 to simulate heavy-tailed errors. In all models $x_{2}∼Bernoulli\;\;\left(p_{x_{2}}=0.2\right)$, which is a binary variable that equals one 20% of the time. These models were repeated for the sample sizes (n = {100, 250, 500, 750}). The outcome variable, y, was rounded to the nearest whole number in all models to make it a discrete variable (y∈N), which are common in environmental epidemiology and health research in general. Examples of discrete outcome include SRS scores,^29^ IQ scores,^30^ or rounded measurements.

To ensure that our models represent realistic scenarios in environmental epidemiology, we selected parameter values based on what we observed from regression models created with the MIREC study,^32^ described below. The outcome (y) variable in our models represents SRS scores, which is used to estimate the frequency and intensity of autistic-like behaviors in toddlers.^29^ The $x_{1}$ variable represents a single toxic chemical, either transformed into a binary variable or left as a continuous variable. We set $\beta_{0}$ = 44, $\beta_{1}$ = 0.5, and $\alpha_{y}$ = 5 in all models based on regression analyses using the MIREC data. This represents how the effect of a single chemical on an outcome is often quite small (relative to the variance of the outcome).^33^ We set $\alpha_{x_{1}}$ = 0.5 so our models will have a similar degree of heteroscedasticity (i.e., difference in associations at the 0.1st vs. the 0.9th quantiles) as the relationship between gestational blood-lead concentrations and SRS scores, as observed in previous research with the MIREC data.^11^ Next, the binary $x_{2}$ variable represents low socioeconomic status, which confounds the relationship between $x_{1}$ and y. We set γ = 0.1 and $\beta_{2}$ = 2.5 based on regression models using the MIREC data. This represent how low socioeconomic status is often associated with higher exposure to some chemicals^34,35^ and diminished child neurodevelopmental outcomes.^36,37^

To further explore our research question, we also considered supplemental models:


$$\begin{array}{lll} y_{i}= & \beta_{0}+\beta_{1}x_{1_{i}}+\beta_{2}x_{2_{i}}+\left(\alpha_{y}+\alpha_{x_{1}}x_{1_{i}}\right)\epsilon_{i};\; & x_{1}∼Discrete\left(\left\{0,\;1,\;2,3\right\},\;p\right);\;\epsilon ∼N\left(0,1\right) \end{array}$$ (6)

$$\begin{array}{lll} y_{i}= & \beta_{0}+\beta_{1}x_{1_{i}}+\beta_{2}x_{2_{i}}+\left(\alpha_{y}+\alpha_{x_{1}}x_{1_{i}}\right)\epsilon_{i};\; & x_{1}∼Discrete\left(\left\{0,\;1,\;2,3\right\},\;p\right);\;\epsilon ∼t\left(4\right) \end{array}$$ (7)

Here, $x_{1}$ is a discrete variable that can take on values of 0, 1, 2, or 3. This denotes a chemical that was transformed such that a one-unit increase denotes a one-quartile increase in chemical concentrations, similar to popular mixture methods like quantile g-computation.^38^ In models 6 and 7, the probability of being assigned a value of {0,1,2,3} is p = (0.25 – γ, 0.25 – $\frac{1}{2}\gamma$, 0.25 + $\frac{1}{2}\gamma$, 0.25 + γ) = (0.15, 0.2, 0.30, 0.35) when $x_{2}$ = 1 (low socioeconomic status), and, p = (0.25 + ($\gamma p_{x_{2}}$), 0.25 + ($\frac{1}{2}\gamma p_{x_{2}}$), 0.25 − ($\frac{1}{2}\gamma p_{x_{2}}$), 0.25 − $\left(\gamma p_{x_{2}}\right)$) = (0.27, 0.26, 0.24, 0.23) when $x_{2}$ = 0 (higher socioeconomic status). This allows p = (0.25, 0.25, 0.25, 0.25) in the entire sample, and represents how the probability of being in the higher quartiles of chemical exposure is often higher among participants with lower socioeconomic status.^34,35^

### QR methods

We considered three QR methods: “standard” (undithered) frequentist, dithered frequentist, and undithered Bayesian. We used the “dither()” function from the quantreg^31^ package to make our dithered frequentist QR models. This adds a random variable with a uniform distribution of [0, 1) to the discrete y variable.^26^ Dithering was not applied to the Bayesian methods because it uses a Metropolis-Hastings algorithm^28^ instead of the simplex algorithm to calculate point estimates, allowing it to generate a smooth and continuous distribution of point estimates even with a discrete y. Our implementation of Bayesian QR had 10,000 MCMC draws with the first 2000 burned. Trace plots indicated that this was sufficient to ensure convergence (Figure S3; https://links.lww.com/EE/A382). The Bayesian methods used an asymmetric Laplace distribution for its likelihood function, as this approach is widely used and has appealing mathematical properties.^28,39–41^ We also used the default uninformative prior that came with the BayesQR package. We did not consider other prior distributions as this default prior was recommended by its creators^28^ (so we anticipate that many epidemiologists will use it) and to simplify our analyses.

We also considered two methods for calculating the frequentist method’s confidence intervals: rank-based and xy-bootstrapped (with 1000 bootstraps used). We focused on these methods because previous research suggests that other methods for calculating confidence intervals have poor coverage or generate very similar results to these methods.^42,43^ Undithered frequentist QR with xy-bootstrapped intervals was not run for models 4 and 5 (where $x_{1}$ was continuous) due to software crashes (see Appendix 1.1; https://links.lww.com/EE/A382). Next, we considered two methods for calculating the Bayesian method’s posterior credible intervals: unadjusted and adjusted. We determined that the adjusted intervals that came with the BayesQR package^28^ produced implausibly wide posterior credible intervals (see Appendix 1.2; https://links.lww.com/EE/A382), and therefore wrote our own function (see Appendix 2.1; https://links.lww.com/EE/A382) for this adjustment using the formulas from page 331 in the study by Yang et al.^44^

### Performance measures

We compared the performance of all the methods with the point estimates ($\hat{\beta_{\tau_{i}}}$), 90% intervals $\left[LB\left(\hat{\beta_{\tau_{i}}}\right),UB\left(\hat{\beta_{\tau_{i}}}\right)\right]$, and the 90% interval widths ($\hat{W\left(\beta_{\tau_{i}}\right)}=UB\left(\hat{\beta_{\tau_{i}}}\right)-LB\left(\hat{\beta_{\tau_{i}}}\right)$) generated across the same N = 1000 simulations. We estimated the bias, empirical standard error of point estimates, empirical coverage probabilities of the 90% intervals, and empirical standard deviation of 90% interval widths using the respective formulas:^45^

$$\begin{array}{l} Bias=\frac{1}{N}\overset{N}{\underset{i=1}{\sum }}\left(\hat{\beta_{\tau_{i}}}-\beta_{\tau }\right) \end{array}$$ (9)

Empirical standard error of point estimates from the given method =

$$\begin{array}{l} \hat{EmpSE}=\sqrt{\frac{1}{N-1}\overset{N}{\underset{i=1}{\sum }}{\left(\hat{\beta_{\tau_{i}}}-\bar{\beta_{\tau }}\right)}^{2}} \end{array}$$ (10)

$$\begin{array}{lll} & Relative\;\%\;dif\;ference\;in\;variance\; & =100\left({\left(\frac{{\hat{EmpSE}}_{method}}{{\hat{EmpSE}}_{ref.}}\right)}^{2}-1\right). \end{array}$$ (11)

Where ${\hat{EmpSE}}_{ref.}$ is the empirical standard error in undithered frequentist QR’s point estimates, and ${\hat{EmpSE}}_{method}$ is the empirical standard error in dithered frequentist or Bayesian QR’s point estimates.

$$\begin{array}{lll} & Empirical\;coverage\;probability\; & =\frac{1}{N}\overset{N}{\underset{i=1}{\sum }}\left(LB\left(\hat{\beta_{\tau_{i}}}\right)\leq \beta_{\tau }\leq UB\left(\hat{\beta_{\tau_{i}}}\right)\right) \end{array}$$ (15)

$$\begin{array}{lll} & Empirical\;standard\;deviation\;of\;90\;\%\;intervals\; & =\sqrt{\frac{1}{N-1}\overset{N}{\underset{i=1}{\sum }}{\left(\hat{W\left(\beta_{\tau_{i}}\right)}-\bar{W\left(\beta_{\tau }\right)}\right)}^{2}}. \end{array}$$ (16)

Here, $\beta_{\tau }$ represents the true parameter value for the effect of $x_{1}$ on y ($\beta_{\tau }$ values for each model and τ are itemized in Table S2; https://links.lww.com/EE/A382). The quantity $\bar{\beta_{\tau }}$ represents the average point estimate across N simulations ($\bar{\beta_{\tau }}=\frac{1}{N}\overset{N}{\underset{i=1}{\sum }}\left(\hat{\beta_{\tau_{i}}}\right)$). Similarly, $\bar{W\left(\beta_{\tau }\right)}$ represents the average interval width across N simulations ($\bar{W\left(\beta_{\tau }\right)}=\frac{1}{N}\overset{N}{\underset{i=1}{\sum }}\hat{W\left(\beta_{\tau_{i}}\right)}$). Simulations that gave infinite-length intervals (which rarely occurred with the undithered rank-based method) were discarded from equations 4 and 5 (see Appendix 1.3; https://links.lww.com/EE/A382). In rare cases where the Bayesian method failed to converge or give a valid point estimates or intervals, we re-ran the Bayesian model up to 20 times. If, for a given simulation, no valid point estimates or intervals were generated after 20 attempts, then we excluded that simulation from equations 1 to 5 (see Appendix 1.4; https://links.lww.com/EE/A382).

### The MIREC study

We applied the QR methods described above to the MIREC study. It is a pan-Canadian pregnancy and birth cohort that recruited 1983 participants from 2008 to 11. See Arbuckle et al.^32^ for further details. We measured first-trimester whole blood concentrations of arsenic, cadmium, lead, and mercury.^11,46^ We considered two transformations of these metals: assigning all $>Q_{50}$ (median) concentrations a 1 and assigning all $\leq Q_{50}$ concentrations a 0 (making metal concentrations a binary variable, similar to models 1–3), and log_2_-transforming all concentrations (making metal concentrations a continuous variable with an approximately normal distribution, similar to models 4–5). Autistic behaviors were documented in a subset (n = 601) of mother-child dyads using the SRS when the child was 3–4-years old (range: 34–85, mean: 45.3, standard deviation: 6.1).^47^ Higher SRS-2 scores denote a higher degree of communication problems and repetitive/nonreciprocal behaviors.^29^ We emphasize again that SRS-2 scores are discrete, as they can only take on whole number values. We controlled for the following variables: child sex, family income (in CAD: ≤$40,000, $40,001–$80,000, $80,001–$100,000, >$100,000), maternal education (high school or less, college or trade school, undergraduate university degree, graduate university degree), maternal age (< or ≥30), self-identified maternal Race, parity (nulliparous or not), maternal cigarette smoking during pregnancy, and whether the mother lives with their partner. Our Bayesian QR models for the MIREC data used 27,000 MCMC draws with the first 2000 burned.^11^ All analyses were restricted to complete cases (n = 568), which is a potential source of bias.^48^

Health Canada Research Ethics Board and the Institutional Review Boards of Simon Fraser University, and Centre Hospitalier Universitaire Sainte-Justine Research Centre approved the MIREC Study. All participants provided written consent for their own and their children’s participation in this study.

## Results: Performance of QR methods on simulated data with discrete outcomes

**Figure 2.**
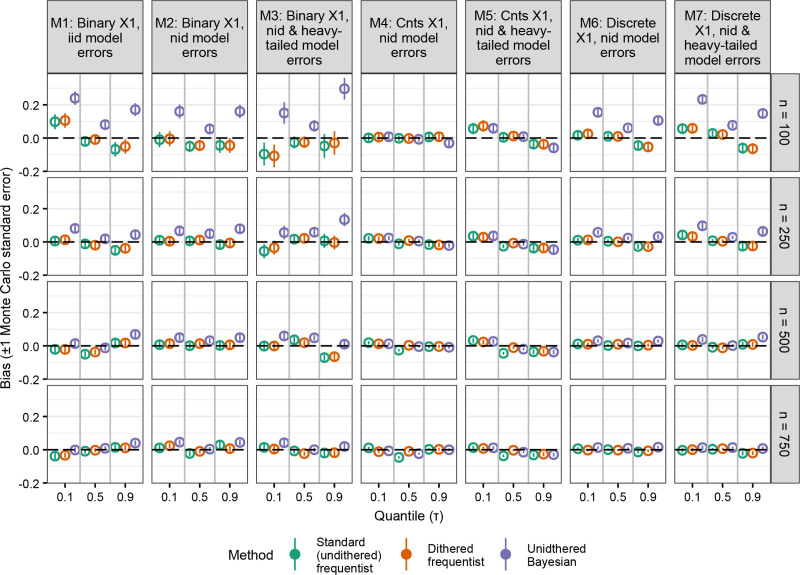
Bias of point estimates from quantile regression methods with a discrete outcome variable. In rare instances, Bayesian quantile regression failed to generate any valid results after 20 attempts. iid indicates independent and identically distributed errors; nid, nonindependently distributed errors.

Undithered and dithered frequentist QR methods both exhibited no or very little bias in all the scenarios we assessed (Figure 2). The bias these methods exhibited was similar. Bayesian QR, however, was biased when $x_{1}$ was binary (models 1–3) or discrete (models 6 and 7). This was especially apparent when errors were heavy-tailed (models 3 and 7), sample sizes were lower, and more extreme τ s (τ = {0.1, 0.9}) were modeled. However, Bayesian QR was unbiased when $x_{1}$ was continuous (models 4 and 5) (Figure 2).

**Figure 3.**
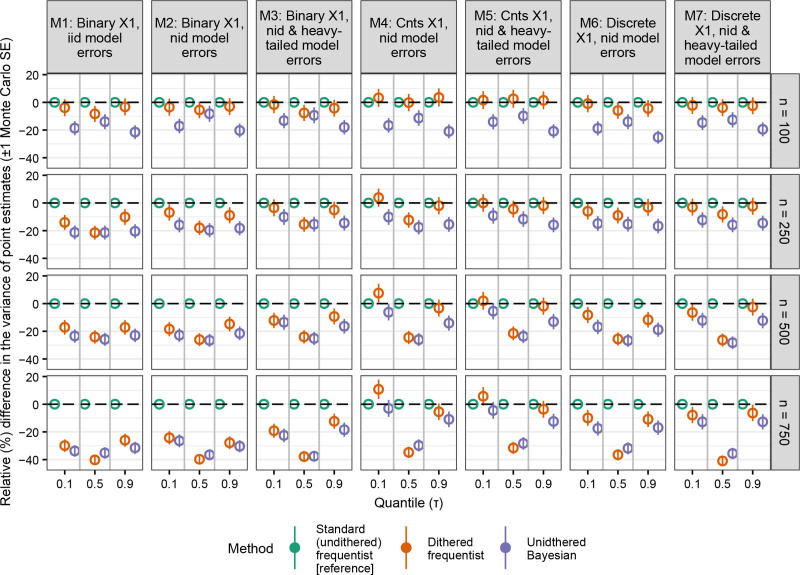
Relative differences in the precision of point estimates from quantile regression methods (with standard undithered frequentist quantile regression as the reference) with a discrete outcome variable. In rare instances, Bayesian quantile regression failed to generate any valid results after 20 attempts. iid indicates independent and identically distributed errors; nid, nonindependently distributed errors.

Undithered frequentist QR’s point estimates were the most variable. Dithered frequentist QR’s point estimates were 5%–40% less variable when $x_{1}$ was binary (models 1–3), and this trend was especially apparent as the sample size increased (Figure 3). Similar results were observed when $x_{1}$ was discrete (models 6 and 7). The difference was less clear when $x_{1}$ was continuous (models 4 and 5), but dithered frequentist QR was 20%–30% less variable when the sample size was larger (n = {500, 750}) and the median (τ = 0.5) was modeled. Next, Bayesian QR generated the least variable point estimates when n = 100, but Bayesian QR and dithered frequentist QR’s point estimates exhibited similar variability (i.e., within one Monte Carlo standard error) when the sample size was larger (n = {500, 750}) (Figure 3).

**Figure 4.**
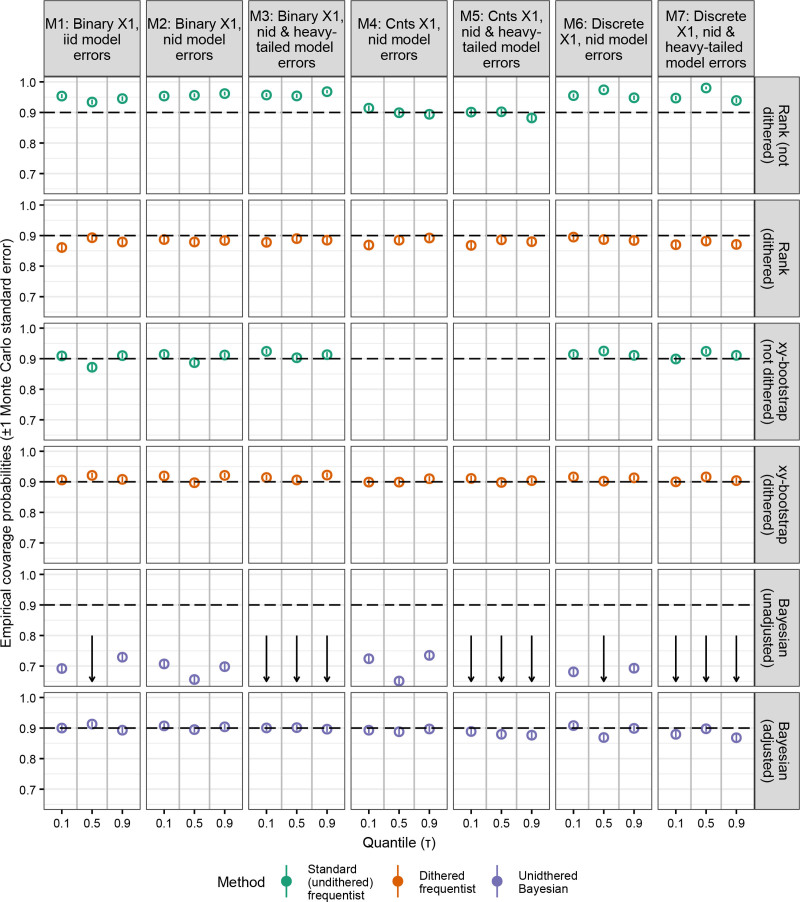
Comparison of the empirical coverage probabilities (circles) of quantile regression methods with a discrete outcome variable. The nominal value (horizontal dashed line) is 0.9. Arrows denote out of range values. Vertical lines depict Monte Carlo standard errors (often very small). A sample size of n = 250 are used throughout. See Figure S4; https://links.lww.com/EE/A382 for results with n = 100, 500, and 750. Simulations with infinitely wide intervals (only observed with the undithered rank-based method) were excluded. The undithered xy method was not run on models 4 and 5 due to software crashes. In rare instances, Bayesian quantile regression failed to generate any valid results after 20 attempts. iid indicates independent and identically distributed errors; nid, nonindependently distributed errors.

Next, Figure 4 presents the empirical coverage probabilities for the 90% confidence or credible intervals with n = 250. Undithered QR with rank-based intervals overcovered when $x_{1}$ was binary (models 1–3) or discrete (models 6 and 7), but had nominal coverage when $x_{1}$ was continuous (models 4 and 5) (Figure 4). Undithered frequentist QR with bootstrap-based intervals had nominal coverage with smaller sample sizes (n = 100 or 250), but not with larger sample sizes (n= {500, 750}). Dithered frequentist QR with both the rank-based and xy-bootstrapped intervals had nominal coverage for all sample sizes and models considered (Figure 4, Figure S4; https://links.lww.com/EE/A382). Next, Bayesian QR with unadjusted intervals had severe undercoverage. Bayesian QR with adjusted intervals had nominal coverage in most scenarios, although the intervals were too narrow when n = 750 and $x_{1}$ was binary (models 1–3) (Figure 4, Figure S4; https://links.lww.com/EE/A382).

**Figure 5.**
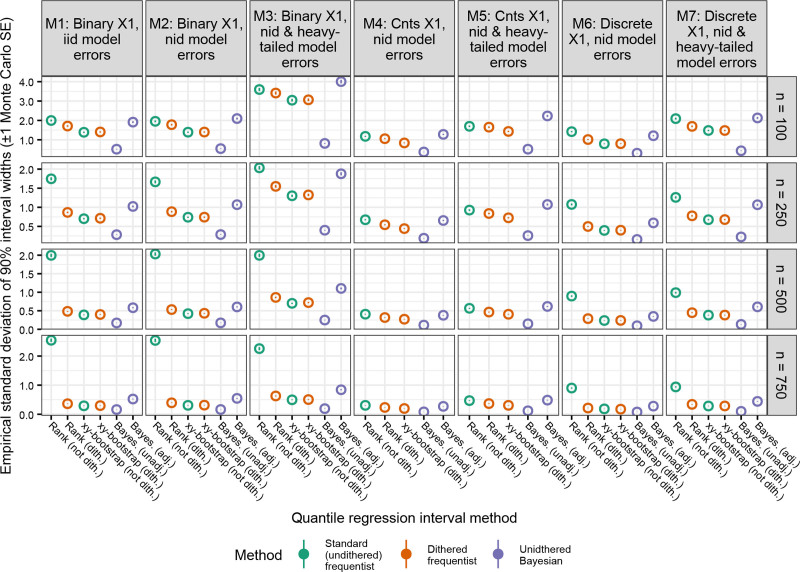
Comparison of the empirical standard deviation of the 90% intervals (circles) from quantile regression methods on a discrete outcome variable. Vertical lines depict Monte Carlo standard errors (often very small). Only the results from the 90th quantile (τ = 0.9) are shown. See Figure S5; https://links.lww.com/EE/A382 for results with τ = 0.1 and 0.5. Simulations with infinitely wide intervals (only observed with the undithered rank-based method) were excluded. The undithered xy method was not run on models 4 and 5 due to software crashes. In rare instances, Bayesian quantile regression failed to generate any valid results after 20 attempts. iid indicates independent and identically distributed errors; nid, nonindependently distributed errors.

Figure 5 presents the variability of the 90% confidence or credible intervals widths produced by each method when τ = 0.9 (see Figure S5; https://links.lww.com/EE/A382 for τ = {0.1, 0.5}). The methods that produced the most variable intervals were undithered QR with rank-based intervals (especially when the sample size was larger) and Bayesian QR with adjusted intervals (Figure 5). When $x_{1}$ was binary (models 1–3) or discrete (models 6 and 7), the variability of the rank-based intervals decreased substantially when dithering was applied, but the improvement was much smaller when $x_{1}$ was continuous (models 4 and 5). The xy-bootstrapped intervals (both dithered and undithered) produced intervals with slightly more consistent widths than the rank-based method. Finally, the unadjusted Bayesian method had the least variable intervals (Figure 5).

Finally, frequentist QR with rank-based intervals had a faster computation time than the bootstrap-based intervals, especially when the sample size was larger. Bayesian QR had by far the slowest computation time. Dithering did not impact computation time (Table S3; https://links.lww.com/EE/A382).

## Discussion

QR allows users to model the relationship between a covariate and an outcome throughout the distribution of the outcome, but it is unclear how to use it when the outcome variable is discrete.^5^ Our simulations with discrete outcomes suggest that dithered frequentist QR (particularly with xy-bootstrapped confidence intervals) had the best overall performance. This method was generally unbiased, produced point estimates that were relatively stable (less variable), and produced confidence intervals with nominal coverage and relatively consistent interval widths (Figures 2–5).

“Standard” (undithered) frequentist QR with discrete outcomes tends to generate implausible point estimates that are integers (i.e., $\beta_{\tau }$ = −2, −1, 0, 1,...) or rational numbers (i.e., $\beta_{\tau }$ = $-\frac{2}{3},\;-\frac{1}{2},\;0,\;\frac{1}{3},\;\frac{1}{2},\;1,\ldots$) (Figure 1 and Table 1). This is not an error, but a consequence of QR’s simplex algorithm for model fitting.^5^ Again, QR works by first calculating the slopes between all covariates and the outcome variable.^5^ QR’s point estimates must be within this set.^5^ When the outcome variable is discrete and all covariates are binary, then all point estimates will be integers or rational numbers (see Figure 1 in models 1–3, where $x_{1}$ and $x_{2}$ are binary). When the outcome variable is discrete and the covariates are a mix of continuous, binary, and/or categorical variables (which is usually the case, as many confounding variables such as education, marital status, sex, and race/ethnicity are noncontinuous), then some point estimates may be integers or rational numbers (see Figure 1 in models 4 and 5, where $x_{1}$ is continuous and $x_{2}$ is binary). Dithering (which adds random noise to the outcome variable) results in a wider range of slopes between datapoints, and therefore a wider range of possible point estimates. Consequently, dithered frequentist QR produced a smooth, normal distribution of point estimates (Figure 1).

The results from our simulation experiment suggest that undithered frequentist QR’s implausible point estimates may result in less reliable inferences for individual studies. When we examine the performance of the point estimates a method generates, there are two considerations: bias and variance. On one hand, undithered frequentist QR exhibited an essentially identical degree of bias compared to dithered frequentist QR (Figure 2) despite generating these implausible point estimates when y was discrete. On the other hand, undithered frequentist QR produced more variable point estimates than dithered frequentist QR. This was most apparent when $x_{1}$ was binary (models 1–3, which always produced integer point estimates, see Figure 1) and when the sample size was higher (which would have permitted point estimates to be estimated to more decimal places had these point estimates not been integers) (Figure 3). This suggests that dithering, which prevents implausible point estimates, produces point estimates that are more reliable for individual studies.

We considered two methods for calculating confidence intervals with frequentist QR when y was discrete: rank-based and bootstrap-based. The rank-based method calculates (1 − α) × 100% intervals by conducting a series of hypotheses in both directions from the point estimate until values that fail to reject at α are identified.^5,49^ When dithering was not applied, the bootstrap-based and especially the rank-based confidence intervals did not have nominal coverage in all the scenarios we considered. Applying dithering resolved this issue (Figure 4), which reinforces the benefits of dithering. Compared to the rank-based method, the bootstrap-based method had less variable interval widths (Figure 5). As a result of the lower and upper bounds being calculated independently,^5^ the rank-based intervals may produce asymmetric intervals (Table 1, Figure S1; https://links.lww.com/EE/A382). Overall, our simulation with discrete outcomes suggest that the rank-based confidence intervals had slightly worse performance than the bootstrap-based confidence intervals despite the rank-based method being the default option in the quantreg package when n <1000.^31^ However, the rank-based method had faster computation time (Table S3; https://links.lww.com/EE/A382), but the difference may not be noticeable on modern computers.

Like dithered frequentist QR, Bayesian QR did not produce implausible point estimates (Figure 1 and Table 1). Our results suggest that Bayesian QR may be biased when estimating the effect of binary covariates, which was especially apparent when the sample size was small (n = 100) (Figure 2). We suspect that this bias may be related to the BayesQR package assuming a normal posterior distribution by default. When the sample size is smaller, the posterior distribution may be more skewed, so the normal approximation of the posterior distribution may be a poor fit. The fact that the Bayesian method’s bias was diminished when modeling a continuous covariate further suggests this. Bayesian QR had the advantage of producing the least variable point estimates when the sample size was small (n = 100) (Figure 3). Next, we found that the Bayesian method’s adjusted intervals had much better coverage than the unadjusted intervals (Figure 4). The adjusted intervals, however, had more variable credible interval widths (Figure 5).

The implausible point estimates that undithered frequentist QR generates with discrete outcomes may be particularly undesirable in environmental epidemiology. In our MIREC example, we found many instances where undithered frequentist QR indicated that there was exactly no association ($\beta_{\tau }$ = 0), but dithered frequentist QR and Bayesian QR estimated a small, but non-zero association ($\beta_{\tau }\neq$ 0) (Table 1). It is therefore possible that undithered frequentist QR can obscure small associations, leading to the mistaken conclusion that no relationship exists. This is a concern as environmental epidemiology is often concerned with estimating small effect sizes. There are several chemicals for which populations are universally or near-universally exposed. Such chemicals can have a substantial impact on the wellbeing of entire populations even if they have a small effect.^50^

Our study has several limitations. Our findings are valid only for discrete outcome variables. Further research on best practices may be needed for ordinal outcome variables.^51^ We did not consider the QR coefficients modeling method, a novel approach that generates a smoothed range of point estimates across the quantiles (τ) of y. This prevents abrupt changes in point estimates from one quantile to the next, which may be more interpretable and increase precision.^52,53^ It is also an alternative to dithering in that it permits reliable inferences with discrete outcome variables.^52,53^ While it is a promising technique, selecting an appropriate model for this method is challenging.^53^ Next, our simulations featured a discrete outcome variable with a moderate degree of dispersion ($\alpha_{y}$ =5). Some discrete outcome variables have less dispersion (i.e., zero-inflated counts), and our simulations may not be applicable to such situations. In these situations, one may wish to use ‘average dithering’, where the dithering process is repeated *m* times and the average $\hat{\beta_{\tau_{i}}}$ and $\hat{SE_{\tau_{i}}}$ values across *m* iterations are used.^26^ This addresses the added variance that dithering introduces,^26^ which may be important when the outcome variable has little variance or the sample size is small. We did not implement average dithering because it is not compatible with rank-based intervals, which does not use standard errors to calculate confidence intervals.^5,49^ Our models only controlled for one covariate (namely $x_{2}$, which represents socioeconomic status). Researchers frequently control for many variables, and this could influence our results. Still, increasing the number of covariates would dramatically increase the run time of our simulations. Finally, we did not consider mixture methods despite their popularity. Software packages for popular mixture methods do not support QR.^38,54,55^

## Conclusions

QR is a useful statistical method that allows users to measure the covariate-outcome relationship throughout the outcome variable’s distribution. It can allow researchers to find associations present at the tails of the outcome variable that would not be identified with conventional means-based OLS methods.^11^ A potential hurdle to a broader adoption of QR is that the nature of frequentist QR means that it can generate an implausible pattern of integer or rational point estimates when the outcome variable is discrete. Our simulation study with discrete outcomes suggests that dithered frequentist QR has better performance than the undithered approach because it produced more precise point estimates, particularly when sample sizes were larger or binary/discrete covariates are modeled (Figure 3). We note that when the outcome variable has a smooth and continuous distribution, dithering (which artificially makes the outcome variable have a smooth and continuous distribution) is not necessary.^26^ Next, the xy-bootstrapped confidence intervals had slightly better performance with discrete outcomes than the default (when n < 1000)^31^ rank-based intervals (Figures 4 and 5). The Bayesian method with adjusted intervals is also an acceptable strategy for discrete outcomes. While our simulations suggest it has some limitations (bias under certain circumstances [Figure 2], and higher interval width variability [Figure 5]), its point estimates were the least variable when the sample size was low (Figure 3).

## ACKNOWLEDGMENTS

We are grateful to all the participants who took part in the Maternal-Infant Research on Environmental Chemicals (MIREC) Study, as well as to all study staff.

### Conflicts of interest statement

The authors declare that they have no conflicts of interest with regard to the content of this report.

## Supplementary Material


