# Supplementary material for: Performance of quantile regression methods with discrete outcomes: A simulation study with applications to environmental epidemiology
Source: Environ Epidemiol. 2025 Oct 28;9(6):e432. doi: 10.1097/EE9.0000000000000432 (PMC12571108; doi:10.1097/EE9.0000000000000432)
Supplement: Supplementary file 1 [file ee9-9-e432-s001.pdf]

Supplemental material for:

Performance of Quantile Regression Methods with Discrete Outcomes: A Simulation Study with Applications to Environmental Epidemiology

Item	Page No.
Table of contents	1
**Appendix 1.** Further explanation of software errors and instances where no valid results were recorded.	2
**Appendix 2.** Select code segments.	6
**Table S1.** Point estimates (and 95% intervals) for the associations between first-trimester metal concentrations and child SRS scores (indicates autistic-like behaviors) using various quantile regression methods, the MIREC Study, Canada, 2008-2011 (n = 568).	11
**Table S2.** True effects (β_τ) of x_1 on y from each model and quantile (τ) we considered.	13
**Table S3.** Computation time for quantile regression methods.	14
**Figure S1.** Point estimates (circles) and 95% intervals (vertical lines) for the associations between first-trimester metal concentrations and child SRS scores (indicates autistic-like behaviors) using various quantile regression methods, the MIREC Study, Canada, 2008-2011 (n = 568).	15
**Figure S2.** Histogram of point estimates from quantile regression methods for all sample size and τ combinations.	17
**Figure S3.** Trace plot for Bayesian quantile regression for all models and sample sizes considered.	22
**Figure S4.** Comparison of the empirical coverage probabilities of quantile regression methods with a discrete outcome variable for n = 100 and 500.	23
**Figure S5.** Comparison of the empirical standard deviation of the 90% intervals from quantile regression methods on a discrete outcome variable for τ = 0.1 and 0.5.	27
**References**	29

Appendix 1. Further explanation of software errors and instances where no valid results were recorded. We experienced various software errors that resulted in us having to exclude some results due to software crashes, infinite intervals, or the Bayesian method failing to give valid results after many attempts. These software errors occurred on a 64-bit Windows 10 computer with R version 4.5.1, quantreg version 6.1,[1] and BayesQR version 2.4.[2] These issues were replicated with a different 64-bit Windows 11 computer with the same software versions.

<u>**1.1**: Issues with undithered frequentist quantile regression and bootstrapped confidence intervals.</u>
When performing simulations of undithered frequentist quantile regression with xy-bootstrapped intervals with a continuous x_1 (models 4 and 5), RStudio would freeze and become unresponsive. This made it impossible to run the undithered xy method on models 4 and 5. After approximately 50 out of the N=1000 iteration, RStudio would freeze and become unresponsive, and this made it not possible to run the simulation for the planned N = 1000 iterations. Setting different seeds resulted in the crash happen at different iterations, but did not prevent the crash. These crashes still occurred when using alternative bootstrap methods in the quantreg package,[1] included weighted xy-bootstrap ('wxy') or Parzen, Wei, and Ying (1994)'s[3] quantile regression bootstrap ('pwy'). Trying different β_0, β_0 , and α_y parameter values also did not resolve this issue, neither did using a lognormal distribution for x_1 instead of a normal distribution. We could not find any information online or in the quantreg vignette that could help us resolve this issue.

Recall that in our simulations, the outcome variable, y, was rounded to the nearest whole number. Dithering (which makes discrete outcomes have an artificially continuous distribution) resolved these crashes. This indicates that these crashes are related to the discreteness of y. Thus, these software crashes may provide further support for our conclusion that dithered frequentist quantile regression should be implemented on discrete outcome.

Finally, we note that it is unlikely that the typical user will encounter this error. We only encountered this error after performing quantile regression many times over, which is typical in Monte Carlo simulation studies, but not in most analyses. As such, it is unlikely that other users will encounter this issue.

<u>**1.2**: Justification for writing our own function for the adjusted Bayesian quantile regression intervals</u>
The BayesQR package can calculate 'adjusted' and 'unadjusted' posterior credible intervals. We did not use this package's adjusted intervals they often produced intervals that were clearly too wide. For 77 out of 84 possible $\tau \times$ n $\times$ model combinations, the BayesQR package's unadjusted 90% posterior credible intervals that covered the true effect **100%** of the time. That is, out of N=1000 simulations, this method's intervals covered the true effect 1000 times. Nominal 90% posterior credible intervals should only cover the true effect 900 times. The lowest coverage rate we observed with BayesQR's unadjusted intervals was 99.8%, which is **far** above the nominal coverage rate.

This result is unprecedented. Yang et al. (2016)'s[4] simulation study suggests that their adjustment to Bayesian quantile regression's credible intervals results in nominal coverage. When using our own function for adjusted Bayesian quantile regression credible intervals that was based on the formulas in Yang et al. (2016)[4] (see Appendix 2.1), we also observed nominal coverage in most of the scenarios we considered. This suggests that there may be a software error preventing the BayesQR package from creating adjusted posterior credible intervals with appropriate widths. Using our own function allowed us to avoid this potential issue and ensure a fair comparison between frequentist and Bayesian quantile regression.

1.3: Rank-based intervals extending to infinity

The rank-based method is known to, on rare occasions, create confidence intervals with infinite widths (see section 3.10.1, page 111 in Koenker 2005, *Quantile Regression*[5]).

Indeed, we observed a small number of instances in our simulation experiment where the rank-based method's upper and/or lower 90% confidence interval bounds extended to infinity. Infinite intervals were only observed when extreme quantiles were modelled (τ = 0.1 or 0.9) or when x_1 was a binary or discrete variable, see the table below.

		Number of instances (percentage) where undithered rank-based 90% intervals extended to infinity						
τ	n	Model 1: **Binary** X1, iid model errors	Model 2: **Binary** X1, nid model errors	Model 3: **Binary** X1, nid & heavy-tailed model errors	Model 4: Continuous X1, nid model errors	Model 5: Continuous X1, nid & heavy-tailed model errors	Model 6: **Discrete** X1, nid model errors	Model 7: **Discrete** X1, nid & heavy-tailed model errors
0.1	100	39 (3.9%)	35 (3.5%)	16 (1.6%)	0 (0%)	0 (0%)	4 (0.4%)	2 (0.2%)
0.5	100	0 (0%)	0 (0%)	0 (0%)	0 (0%)	0 (0%)	0 (0%)	0 (0%)
0.9	100	45 (4.5%)	34 (3.4%)	16 (1.6%)	0 (0%)	0 (0%)	5 (0.5%)	1 (0.1%)
0.1	250	10 (1%)	8 (0.8%)	0 (0%)	0 (0%)	0 (0%)	0 (0%)	0 (0%)
0.5	250	0 (0%)	0 (0%)	0 (0%)	0 (0%)	0 (0%)	0 (0%)	0 (0%)
0.9	250	13 (1.3%)	9 (0.9%)	0 (0%)	0 (0%)	0 (0%)	0 (0%)	0 (0%)
0.1	500	7 (0.7%)	8 (0.8%)	0 (0%)	0 (0%)	0 (0%)	0 (0%)	0 (0%)
0.5	500	1 (0.1%)	0 (0%)	0 (0%)	0 (0%)	0 (0%)	0 (0%)	0 (0%)
0.9	500	10 (1%)	5 (0.5%)	0 (0%)	0 (0%)	0 (0%)	0 (0%)	0 (0%)
0.1	750	22 (2.2%)	12 (1.2%)	0 (0%)	0 (0%)	0 (0%)	0 (0%)	0 (0%)
0.5	750	0 (0%)	0 (0%)	0 (0%)	0 (0%)	0 (0%)	0 (0%)	0 (0%)
0.9	750	19 (1.9%)	11 (1.1%)	0 (0%)	0 (0%)	0 (0%)	0 (0%)	0 (0%)

If a user encounters infinite confidence intervals with the rank-based method, we recommend that they use bootstrapped confidence intervals instead.

1.4: Bayesian quantile regression models that failed to give valid results

We created 84,000 Bayesian quantile regression models during our simulations (3 τs × 4 sample sizes × 6 model × N=1000 simulations). There were rare instances where the model failed to converge or give any valid results. In these cases, we re-ran the model (with the exact same data) up to 20 times to try and obtain valid results. The table below describes the number of times that the Bayesian quantile regression models had to be re-run to achieve valid results.

Number of repeats needed for convergence	Number of instances (%)
0 (worked on first attempt)	82,989 (98.80%)
1	324 (0.39%)
2	6 (0.01%)
3 to 20	0 (0%)
Failed to converge after 20 attempts	681 (0.81%)

Out of the 84,000 models, there were 82,989 (98.80%) instances where the model successfully converged on the first attempt. There were 324 (0.39%) and 6 (0.01%) instances where the model successfully converged after 1 or 2 repeats, respectively. Curiously, there were **no** instances where the model successfully converged after 3 to 19 repeats. There were 681 (0.81%) instances where no valid results were generated after 20 repeats. In these instances, there were no results that could be stored.

In an earlier version of the simulation, we repeated the Bayesian quantile regression model up to 100 times, but even then we were unable to get valid results. It seems that if convergence is not achieved after two repeats, it will not be achieved after a reasonable number of repeats. We also increased the number of Markov Chain Monte Carlo (MCMC) draws from 10,000 to 15,000, but this still did not solve this issue. In our final version of the simulation experiment, we used 10,000 MCMC draws and repeated the Bayesian quantile regression model up to 20 times to decrease the computation demand.

Bayesian quantile regression only failed to converge after 20 attempts in models that featured heavy-tailed errors (models 3, 5, 7). This issue also occurred more frequently when the sample size was higher, see the table below.

		Number of instances (percentage) where Bayesian quantile regression models failed to converge after 20 attempts						
τ	n	Model 1: Binary X1, iid model errors	Model 2: Binary X1, nid model errors	Model 3: Binary X1, nid & **heavy-tailed** model errors	Model 4: Continuous X1, nid model errors	Model 5: Continuous X1, nid & **heavy-tailed** model errors	Model 6: Discrete X1, nid model errors	Model 7: Discrete X1, nid & **heavy-tailed** model errors
0.1	100	0 (0%)	0 (0%)	4 (0.4%)	0 (0%)	4 (0.4%)	0 (0%)	1 (0.1%)
0.5	100	0 (0%)	0 (0%)	4 (0.4%)	0 (0%)	4 (0.4%)	0 (0%)	1 (0.1%)
0.9	100	0 (0%)	0 (0%)	4 (0.4%)	0 (0%)	4 (0.4%)	0 (0%)	1 (0.1%)
0.1	250	0 (0%)	0 (0%)	6 (0.6%)	0 (0%)	11 (1.1%)	0 (0%)	13 (1.3%)
0.5	250	0 (0%)	0 (0%)	6 (0.6%)	0 (0%)	11 (1.1%)	0 (0%)	13 (1.3%)
0.9	250	0 (0%)	0 (0%)	6 (0.6%)	0 (0%)	11 (1.1%)	0 (0%)	13 (1.3%)
0.1	500	0 (0%)	0 (0%)	20 (2%)	0 (0%)	25 (2.5%)	0 (0%)	26 (2.6%)
0.5	500	0 (0%)	0 (0%)	20 (2%)	0 (0%)	25 (2.5%)	0 (0%)	26 (2.6%)
0.9	500	0 (0%)	0 (0%)	20 (2%)	0 (0%)	25 (2.5%)	0 (0%)	26 (2.6%)
0.1	750	0 (0%)	0 (0%)	39 (3.9%)	0 (0%)	27 (2.7%)	0 (0%)	51 (5.1%)
0.5	750	0 (0%)	0 (0%)	39 (3.9%)	0 (0%)	27 (2.7%)	0 (0%)	51 (5.1%)
0.9	750	0 (0%)	0 (0%)	39 (3.9%)	0 (0%)	27 (2.7%)	0 (0%)	51 (5.1%)

If a user encounters the same issue with Bayesian quantile regression failing to generate any results, they should try re-running their model (see Appendix 2.2 for code). If a user fails to generate any valid results after multiple attempts, they should consider increasing the number of MCMC draws and/ or the number of "burned" draws. If that does not work, then they should consider implementing transformations of the outcome variable and/ or the covariates to avoid a heavy-tailed residual distribution. If that is not possible or appropriate, then users should consider using the frequentist method.

Appendix 2. Select code segments.
See https://github.com/jalam11/QR-with-discrete-outcomes for all code. Notable sections of code are included below.

<u>**2.1:** Code for adjusted Bayesian quantile regression credible intervals.</u>

```
summary_sw <- function(object, # the BayesQR object (made from running BayesQR(y ~ x1 + x2 + ...)).
                    ## The chemical/ exposure variable you are interested in must be listed as the **first**
covariate in the BayesQR model
                    n, # the sample size
                    covariates= x, # A vector containing the covariate names, in the same order as the
BayesQR() object
                    burnin = 1,  # The initialy number of iterations burned
                    level = 0.95) { # The confidence interval level

  z <- qnorm( 1 - ((1-level) / 2) )

  tau <- object[[1]]$quantile

  D1.m1 <- sqrt(n)*var(object[[1]]$beta[-(1:burnin),])/1  # from page 331 of Yang. "D1.m1" means "D1 to
the minus 1; which is the inverse of D1"
  X <- covariates ## build an object called "X", which is required
  D0 <- (1/n)*(t(X) %*% X) # from last line on page 331 of Yang et al.

  tmp <- sqrt(diag( tau*(1-tau) * ((D1.m1 %*% D0) %*% D1.m1))) # from last line on page 331 of Yang et
al.

  mn <- mean(object[[1]]$beta[-(burnin),2])  # regression coefficient for chemical (posterior mean)
  se.adj <- tmp[2]
  lower_bound <- mn - se.adj*z
  upper_bound <- mn + se.adj*z

  res <- c(mn, lower_bound, upper_bound)
  return(res)
}
```

__**2.2:** Code for repeating Bayesian quantile regression models__

```
## Bayesian model

repeats_bqr <- 0
repeat{ # Remake model again if model fails to converge

  # Make model
  sink(file="bayesQR.txt") # save lengthy output to a text file so it does not display in console
  bqr_model <- bayesQR(y ~ x1 + x2, quantile = tau, ndraw = ndraw)
  sink()

  # Check if it converged
  model_converged <- ifelse(mean(bqr_model[[1]]$beta[,2]) == "NaN",
                    F, T)

  if(model_converged == T){ # if the model converged

    # Store the models parameters

    ## Bayesian, unadjusted
    bayesQR_coef <- summary(bqr_model,
                    burnin = burnin, credint = c(z_lower, z_upper))[[1]]$betadraw[2,]
    bayes_unadj <- c(bayesQR_coef[1], # beta
                bayesQR_coef[2], # lb
                bayesQR_coef[3]) # ub

    ## Bayesian, adjusted (v1)
    bayes_adjv1 <- c(bayesQR_coef[1], # beta
                bayesQR_coef[4], # adjusted lb
                bayesQR_coef[5]) # unadjusted ub

    ## Bayesian, adjusted (v2)
    bayes_adj_sw <- summary_sw(bqr_model, n = n, burnin = burnin,
                     covariates= as.matrix(cbind(1, x1, x2)), level =level)
    bayes_adjv2 <- c(bayes_adj_sw[1], # beta
                bayes_adj_sw[2], # lb
                bayes_adj_sw[3]) # ub

    break # end the repeat statement
  }
```

```
    if(model_converged == F){ # if the model fails to converge
      repeats_bqr <- repeats_bqr + 1
      print(paste0("bqr Repeat # ", repeats_bqr))
    }

    if(repeats_bqr == 20){ # if model fails to converge too many times
      print(paste0("Model failed to converge too many times, will assign NA to BayesQR models for
model#", model,
                   ", n=", n, ", tau=", tau, ", iteration #", i))

      ## Bayesian, unadjusted
      bayes_unadj <- c(NA, NA, NA) # assign NA

      ## Bayesian, adjusted (v1)
      bayes_adjv1 <- c(NA, NA, NA) # assign NA

      ## Bayesian, adjusted (v2)
      bayes_adjv2 <- c(NA, NA, NA) # assign NA

      break # end the repeat statement
    }
  }
```

<u>**2.3:** Code for creating simulated models</u>

```
# For creating 90% Confidence intervals. This can be adjusted if you want to create 95% confidence
intervals
z <- qnorm(0.95); z_lower <- 0.05; z_upper <- 0.95; level = 0.9

## x2
prob_x2 <- 0.2 # probability that the confounder x2 equals 1

## x1
### binary
gamma <- 0.1 # effect of x2 on x1
prob_x1_binary <- 0.5 - (gamma * prob_x2) # probability that binary x1=1 should be 50% after
accounting for confounders

### Continuous
mu_x1 <- 0 # mean of continuous x1
```

```
sigma_x1 <- 1 # variance of continuous x1

### Categorical
prob_x1_cat <- c(0.25, 0.25, 0.25, 0.25) # Simulates people having equall probability of falling into each category

# But people with lower SES (which is simulated with x2) should have higher probability of having higher exposure
prob_x1_cat_lowSES <- prob_x1_cat + c(-gamma,
                                     -gamma * 0.5,
                                     gamma * 0.5,
                                     gamma)

# And people with higher SES (which is simulated with x2) should have higher probability of having lower exposure
## If prob_x2 = 0.5, then these two sets of probabilities will be the same.
## But because this is not the case, we need to decrease the effect of being low SES on x1 catergory to allow the
## Probability of falling into each of the four categories to be 0.25 overall
prob_x1_cat_highSES <- prob_x1_cat + c(gamma * prob_x2,
                                      gamma * 0.5 * prob_x2,
                                      -gamma * 0.5 * prob_x2,
                                      -gamma* prob_x2)

# Make function for this
make_cat_x1 <- function(n, x2 = x2,
                        prob_lowSES = prob_x1_cat_lowSES,
                        prob_highSES = prob_x1_cat_highSES) {
  prob = ifelse(x2 == 1, # If x2 equals 1
                prob_x1_cat_lowSES, # use x1 probabilities for low SES people
                prob_x1_cat_highSES) # otherwise, use x1 probabilities for high SES people

  # make categorical x1
  x1 <- sample(c(0, 1, 2, 3), size = n, replace = T)
  return(x1)
}


## y parameters
beta0 <- 44 # true y value when x1 and x2 equall 0
beta1 <- 0.5 # true effect of x1 on y
beta2 <- 2.5 # true effect of x2 on y
```

```
mu_u <- 0 # mean model error value
sigma_u <- 1 # variance of model errors
tdist_df <- 4 # Degrees of freedom for the t distribution
alpha_y <- 5 # model error value is multiplied by this

## misc parameters
boot <- 1000 # Number of xy bootstraps selected
ndraw <- 10000 # Number of MCMC draws for Bayesian QR
burnin <- 2000 # Initial number of MCMC draw that are 'burned' or discarded

# Parameters that were adjusted in the simulation experiment
n <- 100 # Sample sizes considered in this paper include 100, 250, 500, 750. You can edit this to whatever you like

alpha_x1 <- 0.5 # Setting this to 0.5 results in heteroscedastic errors. Set to 0 for homoscedastic errors (associations are constant across all taus). Set to >0.5 to model even stronger heteroscedasticity.

# Covariates
x2 <- rbinom(n, 1, prob_x2) # create a confounding variable that is associated with x and y

x1_binary <- rbinom(n, 1, prob_x1_binary + gamma*x2) # binary x1 for models 1, 2, 3, that depends on x2

x1_cnts <- rnorm(n, mu_x1 + gamma*x2, sigma_x1) # Continuous x1 for models 4 and 5, that depends on x2

x1_discrete <- make_cat_x1(n = n, x2 = x2) # create a discrete variable with values ranging from 0:3, that depends on x2

# Model errors
e_normal <- rnorm(n, mu_u, sigma_u) # Normally distributed errors
e_heavy_tailed <- rt(n, df = tdist_df, mu_u) # heavy-tailed (t distribution with df=4) model errors for models 3 and 5

# Discrete outcome variable
## For this version I used a binary x1 and a normally distributed error distribution (e_normal). Feel free to explore different values.
x1 <- x1_binary
e <- e_normal
y <- round(beta0 + beta1*x1 + beta2*x2 + e*(alpha_y + alpha_x1*x1), digits=0)
```

Table S1. Point estimates (and 95% intervals) for the associations[a] between first-trimester metal concentrations and child SRS scores (indicates autistic-like behaviors) using various quantile regression methods, the MIREC Study, Canada, 2008-2011 (n = 568).

Quantile Regression Method	Chemical name	β_τ (95% intervals) τ = 0.1	τ = 0.5	τ = 0.9
Binary chemical concentrations (above versus below median)				
Rank (not dithered)	Arsenic	-0.5000 (-0.9560, 1.2897)	0.2500 (-1.0193, 1.5129)	-0.6667 (-2.5084, 0.9878)
	Cadmium	-1.0000 (-1.0000, 0.6646)	1.0000 (-0.6215, 1.0000)	1.0000 (-0.8748, 3.7520)
	Lead	0.5000 (-0.9416, 1.4345)	0.0000 (-1.1454, 1.5533)	0.3333 (-1.4761, 1.9343)
	Mercury	-0.6667 (-1.4153, 0.7582)	-0.3333 (-1.7160, 0.9206)	-1.3333 (-3.4121, 0.1234)
xy-bootstrap (not dithered)	Arsenic	-0.5000 (-1.5892, 0.5892)	0.2500 (-0.8385, 1.3385)	-0.6667 (-2.7503, 1.4169)
	Cadmium	-1.0000 (-2.0674, 0.0674)	1.0000 (-0.1573, 2.1573)	1.0000 (-1.1428, 3.1428)
	Lead	0.5000 (-0.5901, 1.5901)	0.0000 (-1.3067, 1.3067)	0.3333 (-1.6903, 2.3570)
	Mercury	-0.6667 (-1.8565, 0.5232)	-0.3333 (-1.6110, 0.9444)	-1.3333 (-3.2442, 0.5776)
Rank (dithered)	Arsenic	-0.3162 (-1.1462, 1.1535)	0.4962 (-1.0361, 1.2205)	-0.6729 (-2.4828, 1.2481)
	Cadmium	-0.1566 (-1.1320, 0.8887)	0.7034 (-0.6741, 1.6000)	0.8932 (-0.9564, 3.6444)
	Lead	-0.1327 (-0.9530, 1.3664)	0.1195 (-0.9775, 1.4388)	0.0416 (-1.7129, 1.7562)
	Mercury	-0.5095 (-1.3300, 0.4815)	-0.3711 (-1.4653, 0.8925)	-1.4129 (-2.8419, -0.1503)
xy-bootstrap (dithered)	Arsenic	-0.3162 (-1.4052, 0.7729)	0.4962 (-0.5984, 1.5909)	-0.6729 (-2.7132, 1.3674)
	Cadmium	-0.1566 (-1.2180, 0.9048)	0.7034 (-0.4571, 1.8640)	0.8932 (-1.1641, 2.9505)
	Lead	-0.1327 (-1.2818, 1.0165)	0.1195 (-1.0731, 1.3121)	0.0416 (-1.8470, 1.9301)
	Mercury	-0.5095 (-1.5642, 0.5452)	-0.3711 (-1.6343, 0.8921)	-1.4129 (-3.3268, 0.5010)
Bayesian (unadjusted)	Arsenic	-0.1238 (-0.9248, 0.6601)	0.2804 (-0.3013, 0.8637)	-0.7753 (-1.7708, 0.2731)
	Cadmium	-0.2732 (-1.0250, 0.4715)	0.5297 (-0.0790, 1.1193)	1.2340 (0.1367, 2.3453)
	Lead	0.3505 (-0.4341, 1.1133)	0.1584 (-0.4905, 0.8259)	0.2538 (-0.7325, 1.2767)
	Mercury	-0.2809 (-1.0728, 0.5215)	-0.3149 (-0.9581, 0.3165)	-1.3923 (-2.3334, -0.3920)
Bayesian (adjusted)	Arsenic	-0.1238 (-1.3421, 1.0991)	0.2804 (-0.7801, 1.3386)	-0.7753 (-2.8259, 1.2747)
	Cadmium	-0.2732 (-1.3548, 0.8055)	0.5297 (-0.5712, 1.6293)	1.2340 (-1.0843, 3.5540)
	Lead	0.3505 (-0.8008, 1.4993)	0.1584 (-1.2200, 1.5372)	0.2538 (-1.6682, 2.1708)
	Mercury	-0.2809 (-1.4678, 0.8992)	-0.3149 (-1.5657, 0.9362)	-1.3923 (-3.1316, 0.3479)
Continuous log2-transformed chemical concentrations				
Rank (not dithered)	Arsenic	-0.1990 (-0.8641, 0.6308)	-0.0420 (-0.7908, 0.3053)	0.3153 (-0.8368, 1.3799)
	Cadmium	-0.4042 (-0.8650, 0.2812)	0.0000 (-0.5218, 0.7291)	0.4162 (-0.6869, 1.7989)
	Lead	0.5169 (-0.6441, 1.2583)	0.2014 (-0.6603, 1.3104)	0.4057 (-1.5029, 1.9234)
	Mercury	-0.2146 (-0.6073, 0.2022)	0.0000 (-0.4614, 0.2788)	-0.1916 (-0.7945, 0.4429)
xy-bootstrap (not dithered)	Arsenic	-0.1990 (-0.9481, 0.5501)	-0.0420 (-0.6261, 0.5422)	0.3153 (-1.0542, 1.6849)
	Cadmium	-0.4042 (-0.9700, 0.1616)	0.0000 (-0.5602, 0.5602)	0.4162 (-0.7093, 1.5418)
	Lead	0.5169 (-0.3087, 1.3425)	0.2014 (-0.7482, 1.1511)	0.4057 (-1.2327, 2.0440)
	Mercury	-0.2146 (-0.6222, 0.1931)	0.0000 (-0.3760, 0.3760)	-0.1916 (-0.9004, 0.5171)
Rank (dithered)	Arsenic	-0.2295 (-0.9649, 0.5631)	-0.1194 (-0.7562, 0.4303)	0.1535 (-0.9151, 1.6073)
	Cadmium	-0.4292 (-0.7748, 0.2452)	-0.0145 (-0.4159, 0.7709)	0.3559 (-0.6320, 1.9285)
	Lead	0.5043 (-0.5687, 1.0906)	0.2482 (-0.6724, 1.2712)	0.2076 (-1.1843, 2.0486)
	Mercury	-0.1761 (-0.5767, 0.1180)	-0.0108 (-0.4565, 0.2291)	-0.3408 (-0.9080, 0.5037)

Quantile Regression Method	Chemical name	β_τ (95% intervals)		
		τ = 0.1	τ = 0.5	τ = 0.9
xy-bootstrap (dithered)	Arsenic	-0.2295 (-0.9796, 0.5206)	-0.1194 (-0.7085, 0.4696)	0.1535 (-1.2282, 1.5352)
	Cadmium	-0.4292 (-1.0023, 0.1439)	-0.0145 (-0.5999, 0.5709)	0.3559 (-0.7141, 1.4260)
	Lead	0.5043 (-0.3635, 1.3721)	0.2482 (-0.6760, 1.1725)	0.2076 (-1.4191, 1.8342)
	Mercury	-0.1761 (-0.5400, 0.1877)	-0.0108 (-0.4000, 0.3784)	-0.3408 (-1.0582, 0.3766)
Bayesian (unadjusted)	Arsenic	-0.2238 (-0.6999, 0.2455)	-0.1629 (-0.4933, 0.1501)	0.2065 (-0.3772, 0.7908)
	Cadmium	-0.3964 (-0.7976, 0.0075)	0.0222 (-0.2904, 0.3427)	0.5015 (-0.0486, 1.0431)
	Lead	0.5083 (-0.0438, 1.0527)	0.2982 (-0.2225, 0.8085)	0.7461 (-0.0608, 1.5330)
	Mercury	-0.1801 (-0.4738, 0.1095)	-0.0378 (-0.2382, 0.1539)	-0.2390 (-0.5739, 0.1001)
Bayesian (adjusted)	Arsenic	-0.2238 (-1.0497, 0.6035)	-0.1629 (-0.7838, 0.4579)	0.2065 (-1.0741, 1.4911)
	Cadmium	-0.3964 (-0.9671, 0.1732)	0.0222 (-0.5598, 0.6039)	0.5015 (-0.6222, 1.6294)
	Lead	0.5083 (-0.2334, 1.2500)	0.2982 (-0.8907, 1.4875)	0.7461 (-1.0761, 2.5699)
	Mercury	-0.1801 (-0.6410, 0.2792)	-0.0378 (-0.3825, 0.3076)	-0.2390 (-0.9048, 0.4284)

a: Controls for child sex, family income, maternal education, maternal age, self-identified maternal Race, parity, maternal cigarette smoking during pregnancy, and whether the mother lives with their partner. Point estimates are rounded to four decimal places to demonstrate that many of them are rational numbers or integers.

Abbreviations: MIREC; Maternal-Infant Research on Environmental Chemicals Study; SRS, Social Responsiveness scale.

Table S2. True effects (β_τ) of x_1 on y from each model and quantile (τ) we considered.

	True effect (β_τ)		
Model	τ = 0.1	τ = 0.5	τ = 0.9
1[a]	0.50	0.50	0.50
2[b]	-0.14	0.50	1.14
3[c]	-0.27	0.50	1.27
4[b]	-0.14	0.50	1.14
5[c]	-0.27	0.50	1.27
6[b]	-0.14	0.50	1.14
7[c]	-0.27	0.50	1.27

[a]: The true effect of x_1 on y is equal to β_1, and because model 1 is homoscedastic, the true effect is constant throughout the y distribution. Recall that $\beta_1 = 0.5$.

[b]: The true effect of x_1 on y is equal to $\beta_1 + Z_\tau \times \alpha_{x_1}$ where Z_τ denotes the τth quantile of the error distribution (a normal distribution with $\mu = 0,\ \sigma^2$=1), and where α_{x_1} controls the severity of heteroscedasticity caused by x_1. Recall that $\beta_1 = 0.5$, and $\alpha_{x_1} = 0.5$.

[c]: The true effect of x_1 on y is equal to $\beta_1 + Z_\tau \times \alpha_{x_1}$ where Z_τ denotes the τth quantile of the error distribution (a t-distribution with 4 degrees of freedom, and where α_{x_1} controls the severity of heteroscedasticity caused by x_1. Recall that $\beta_1 = 0.5$, and $\alpha_{x_1} = 0.5$.

Table S3. Computation time[a] for quantile regression methods. Model 1 and τ = 0.5 used throughout.

Method	Mean (SD) computation time, seconds			
	n = 100	n = 250	n = 500	n = 750
Rank (not dithered)	0.01 (0.01)	0.01 (0.02)	0.01 (0.01)	0.01 (0.02)
xy-bootstrap[b] (not dithered)	0.02 (0.04)	0.06 (0.05)	0.15 (0.07)	0.29 (0.09)
Rank (dithered)	0.01 (0.01)	0.01 (0.01)	0.01 (0.01)	0.01 (0.03)
xy-bootstrap[b] (dithered)	0.02 (0.03)	0.06 (0.03)	0.15 (0.07)	0.29 (0.10)
Bayesian[c] (unadjusted)	0.55 (0.06)	1.22 (0.09)	2.34 (0.20)	3.48 (0.47)
Bayesian[c] (adjusted)	0.55 (0.05)	1.22 (0.09)	2.33 (0.19)	3.47 (0.47)

a: With a Windows 10 computer, i7-7700 processor, R version 4.5.1.

b: 1000 bootstraps used. Computation time depends on the number of bootstraps used.

c: 10,000 Markov Chain Monte Carlo iterations used. Computation time depends on the number of iterations used.

Figure S1. Point estimates (circles) and 95% intervals (vertical lines) for the associations between first-trimester metal concentrations and child SRS scores (indicates autistic-like behaviors) using various quantile regression methods, the MIREC Study, Canada, 2008-2011 (n = 568). Controls for child sex, family income, maternal education, maternal age, self-identified maternal Race, parity, maternal cigarette smoking during pregnancy, and whether the mother lives with their partner.

Abbreviations: MIREC; Maternal-Infant Research on Environmental Chemicals Study; SRS, Social Responsiveness scale.

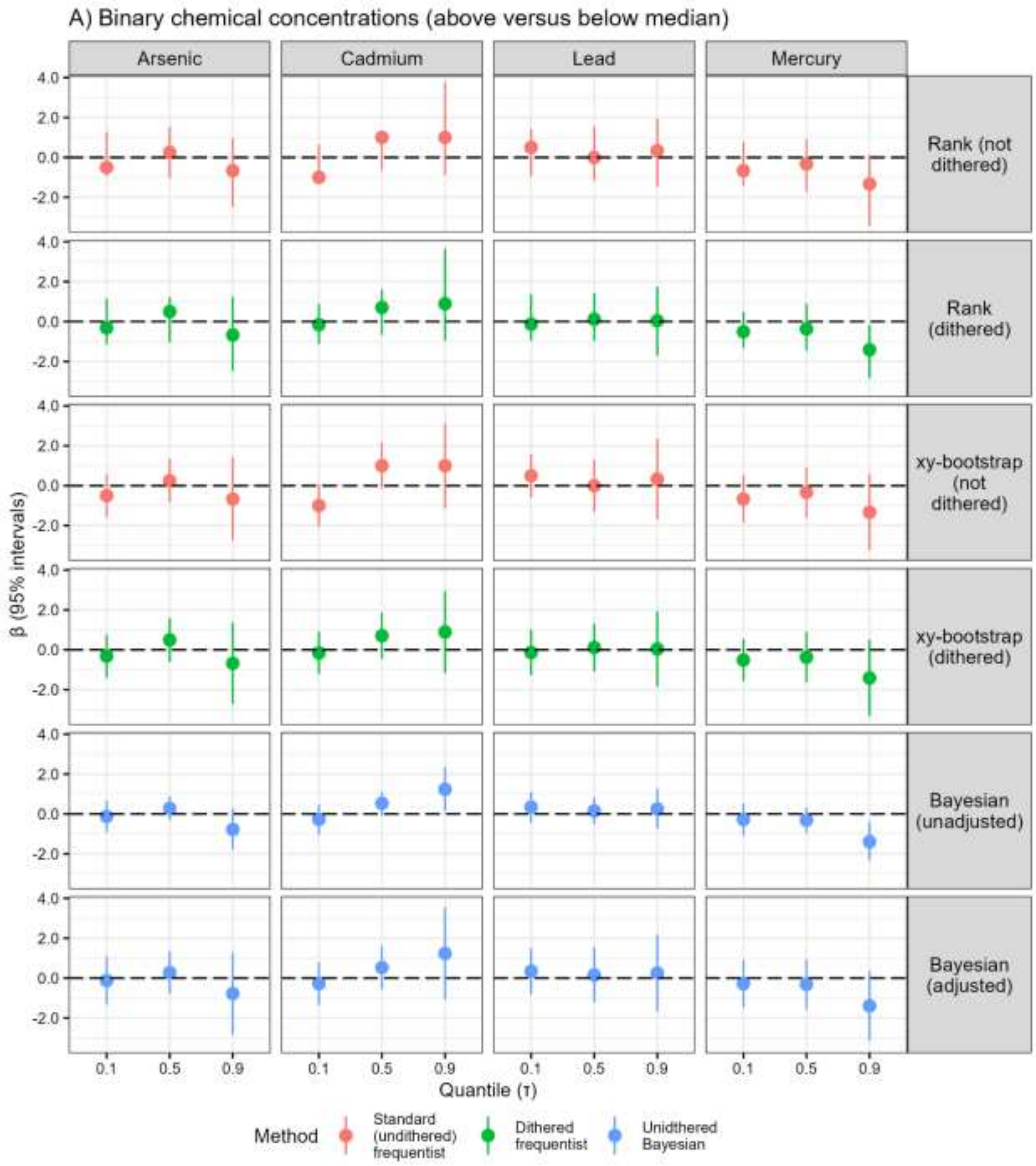

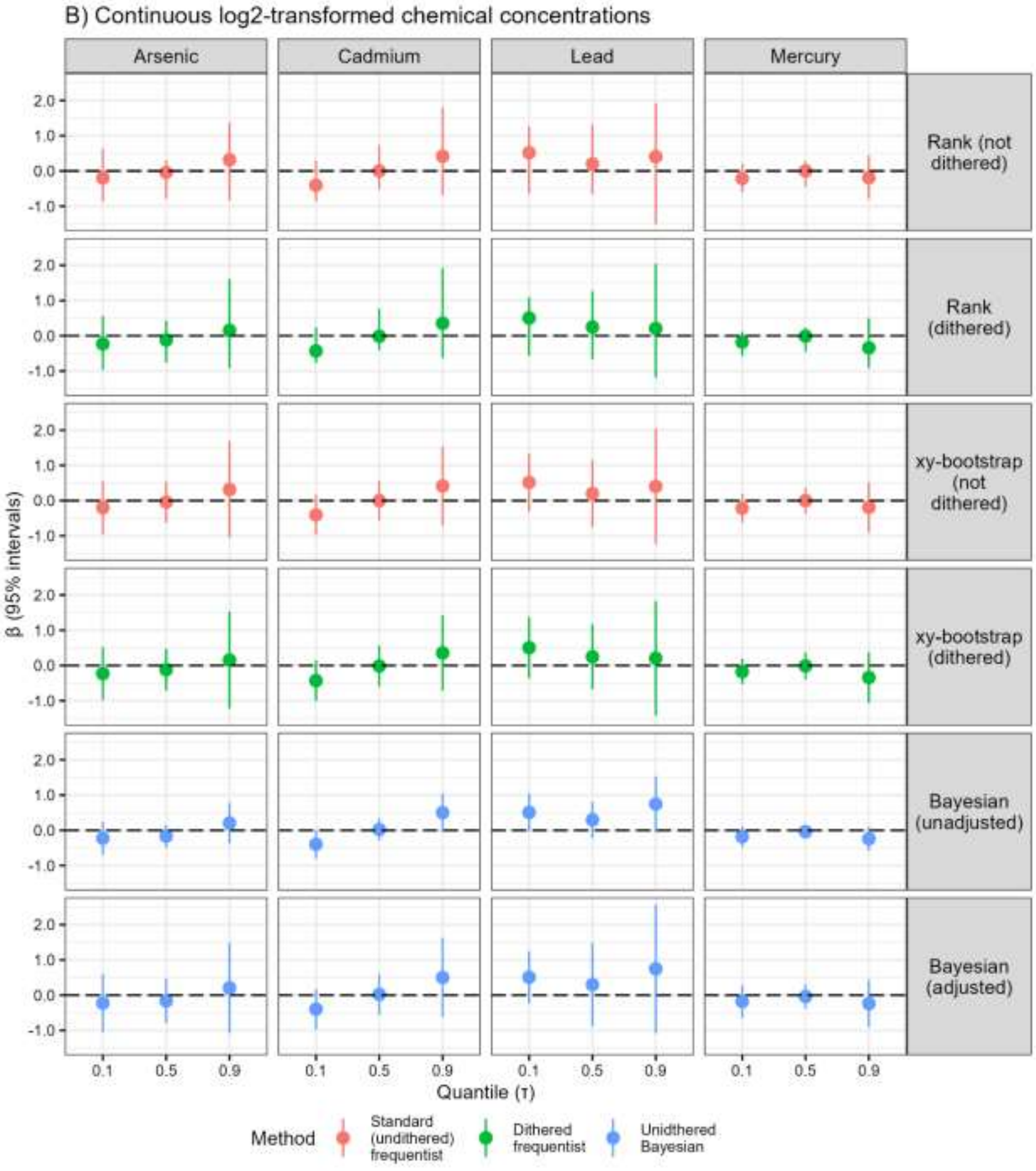

B) Continuous log2-transformed chemical concentrations
Arsenic
Cadmium
Lead
Mercury
Rank (not dithered)
Rank (dithered)
xy-bootstrap (not dithered)
xy-bootstrap (dithered)
Bayesian (unadjusted)
Bayesian (adjusted)
β (95% intervals)
2.0
1.0
0.0
-1.0
0.1
0.5
0.9
Quantile (τ)
Method
Standard (undithered) frequentist
Dithered frequentist
Unidthered Bayesian

Figure S2. Histogram of point estimates from quantile regression methods for all sample size and τ combinations. The vertical dashed lines denote the true effect of x_1 on y.

Abbreviations: iid, independent and identically distributed errors; nid, non-independently distributed errors.

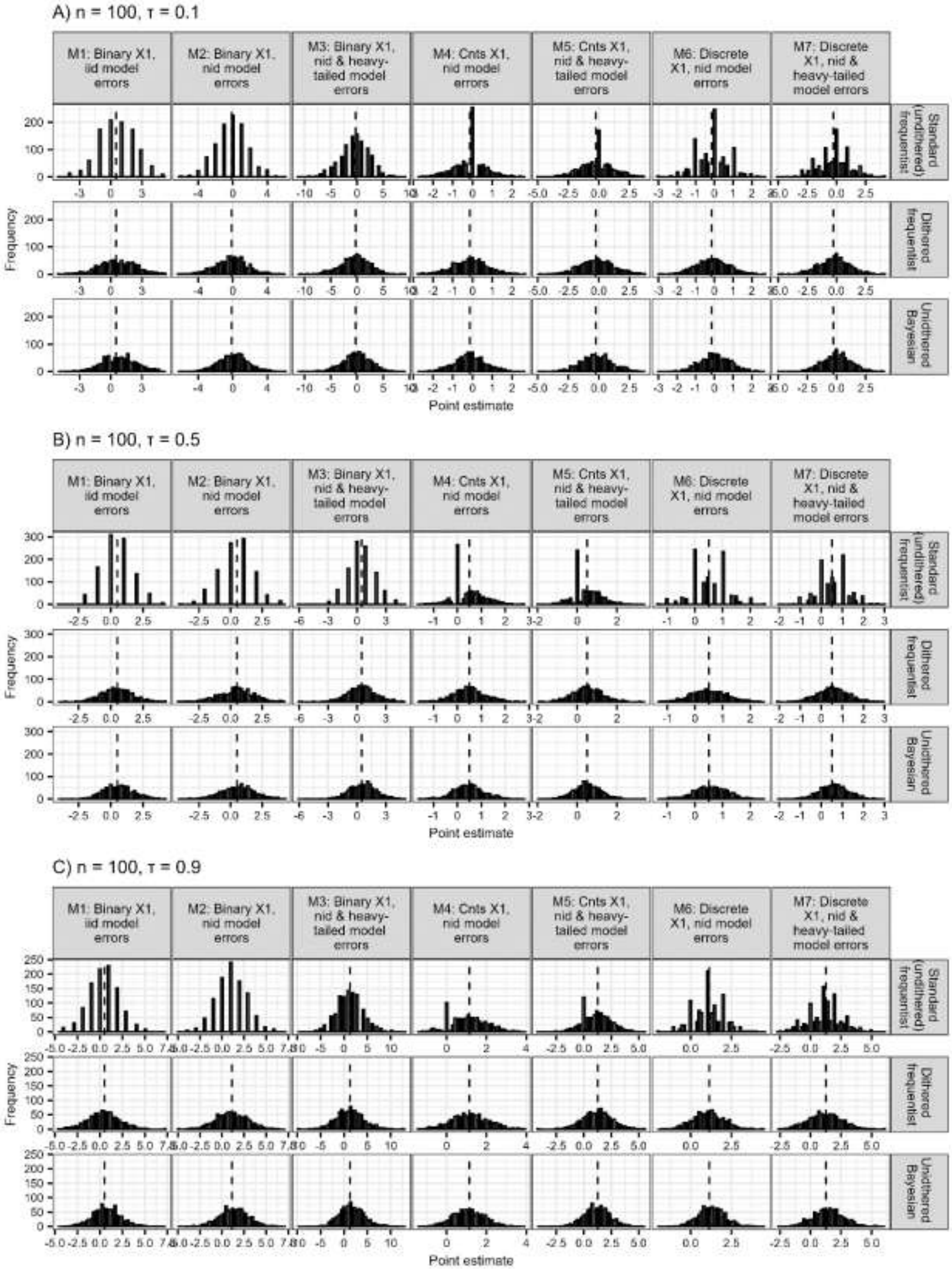
A) n = 100, τ = 0.1
B) n = 100, τ = 0.5
C) n = 100, τ = 0.9
M1: Binary X1, iid model errors
M2: Binary X1, nid model errors
M3: Binary X1, nid & heavy-tailed model errors
M4: Cnts X1, nid model errors
M5: Cnts X1, nid & heavy-tailed model errors
M6: Discrete X1, nid model errors
M7: Discrete X1, nid & heavy-tailed model errors
Standard (undithered) frequentist
Dithered frequentist
Undithered Bayesian
Frequency
Point estimate

D) n = 250, τ = 0.1

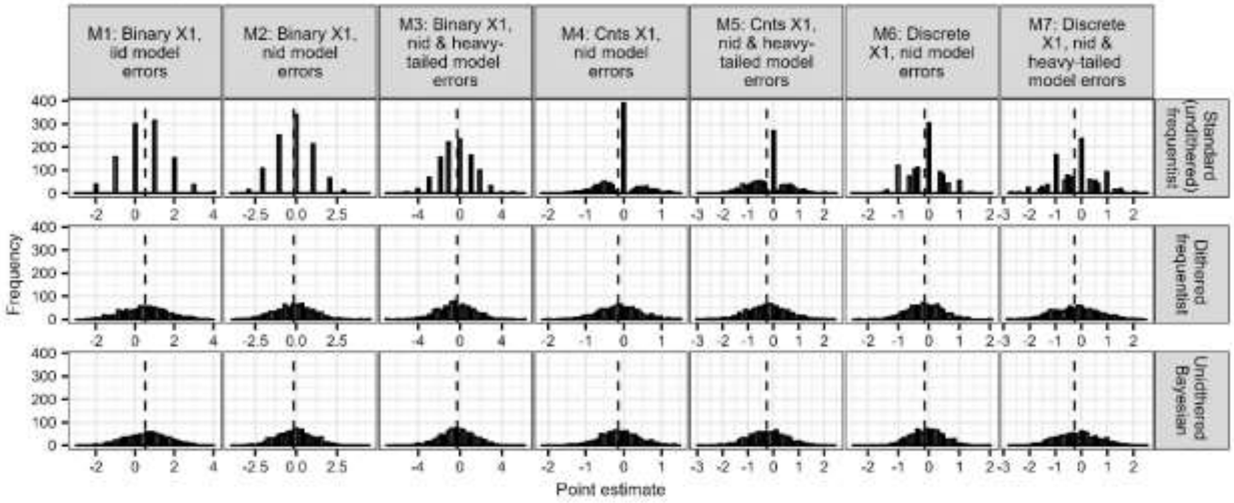

E) n = 250, τ = 0.5

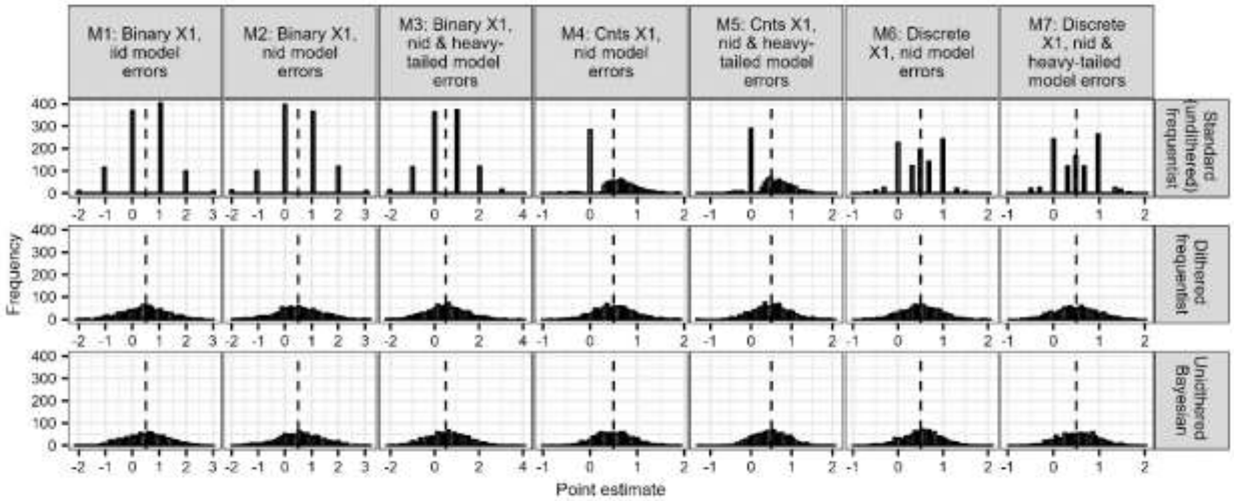

F) n = 250, τ = 0.9

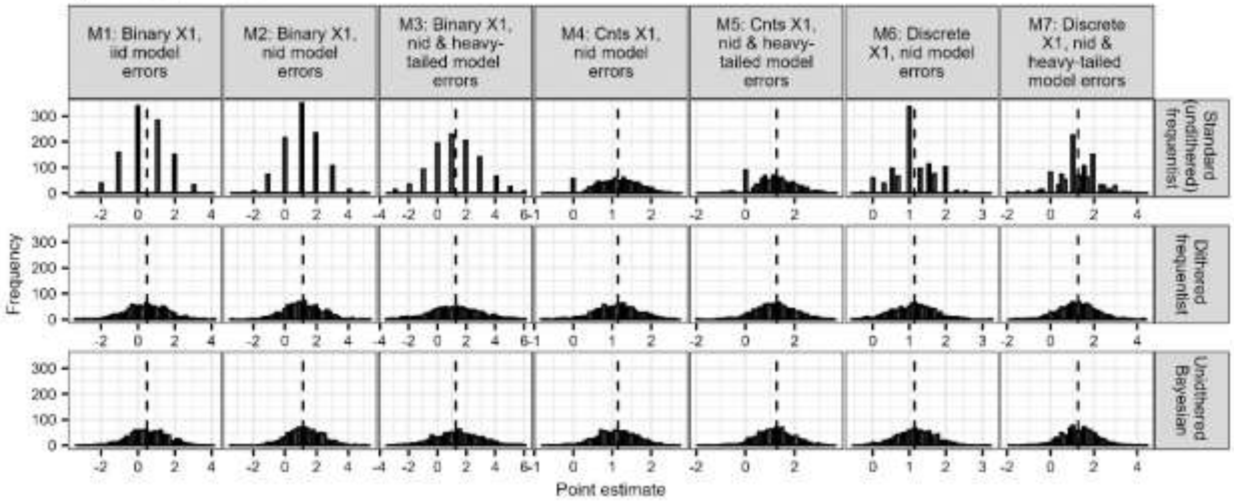

G) n = 500, τ = 0.1

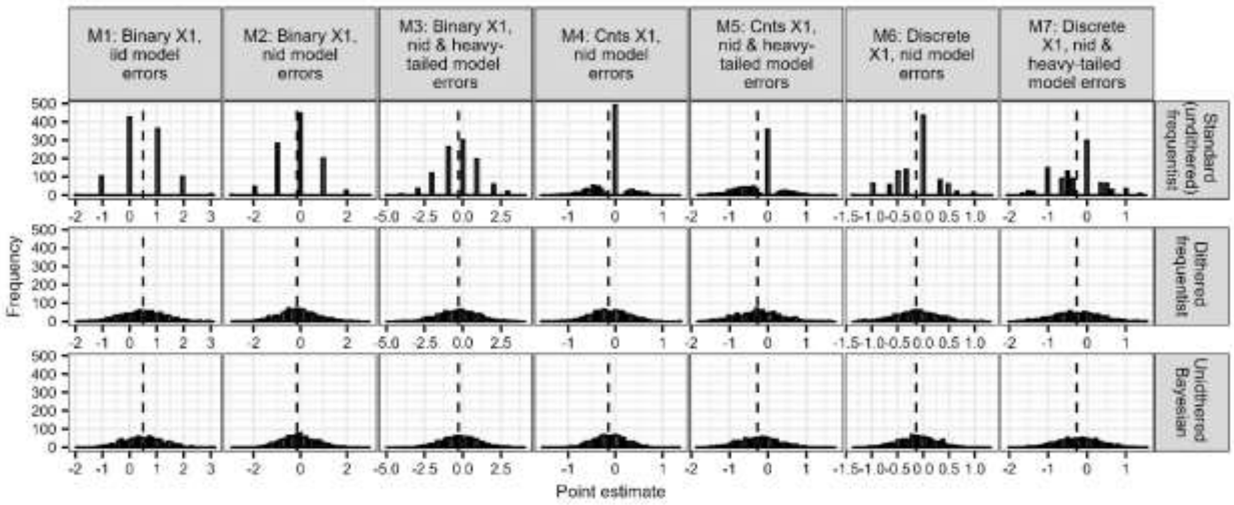

H) n = 500, τ = 0.5

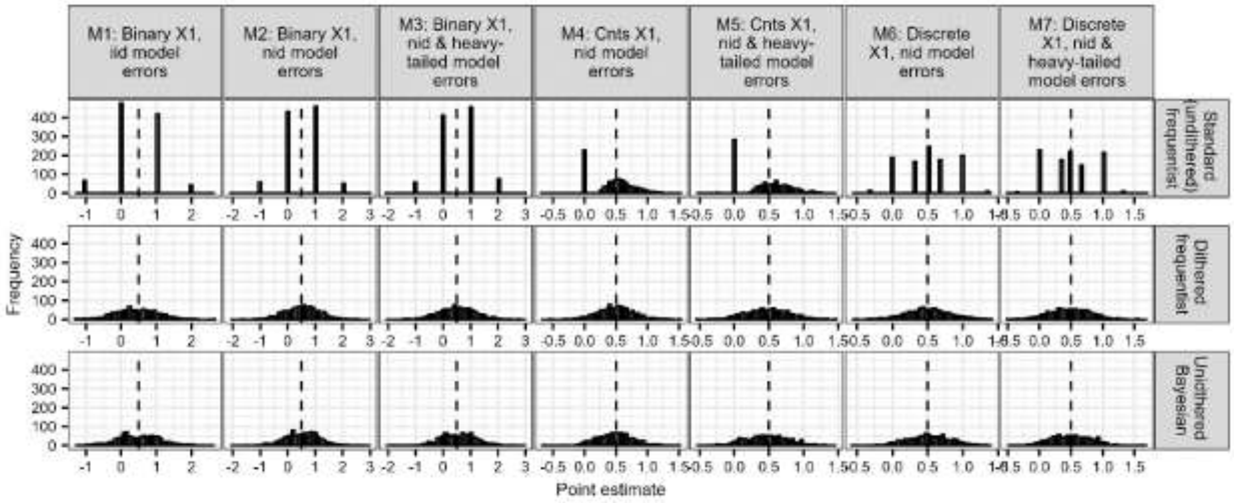

I) n = 500, τ = 0.9

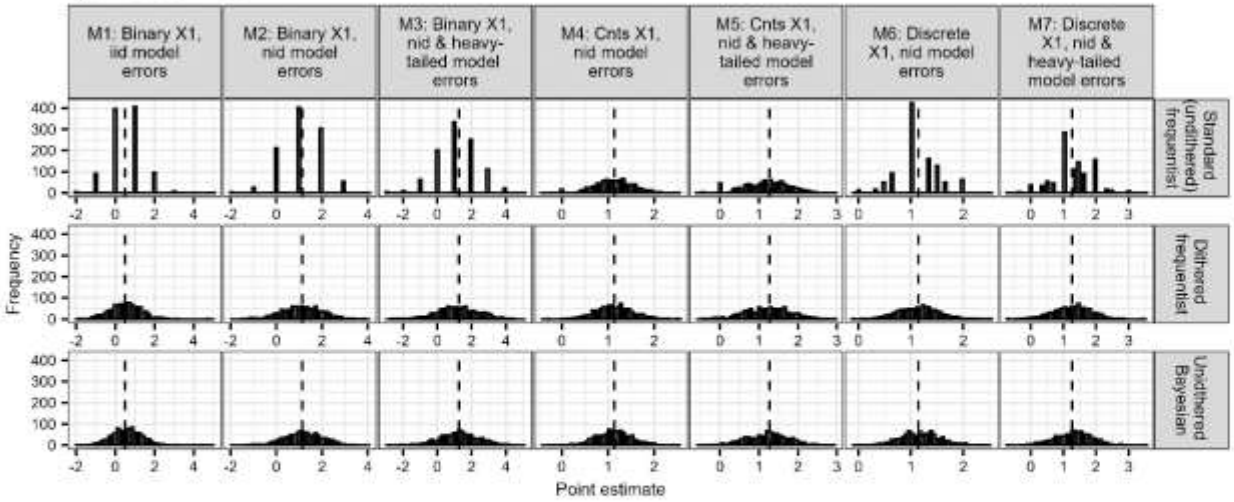

J) n = 750, τ = 0.1

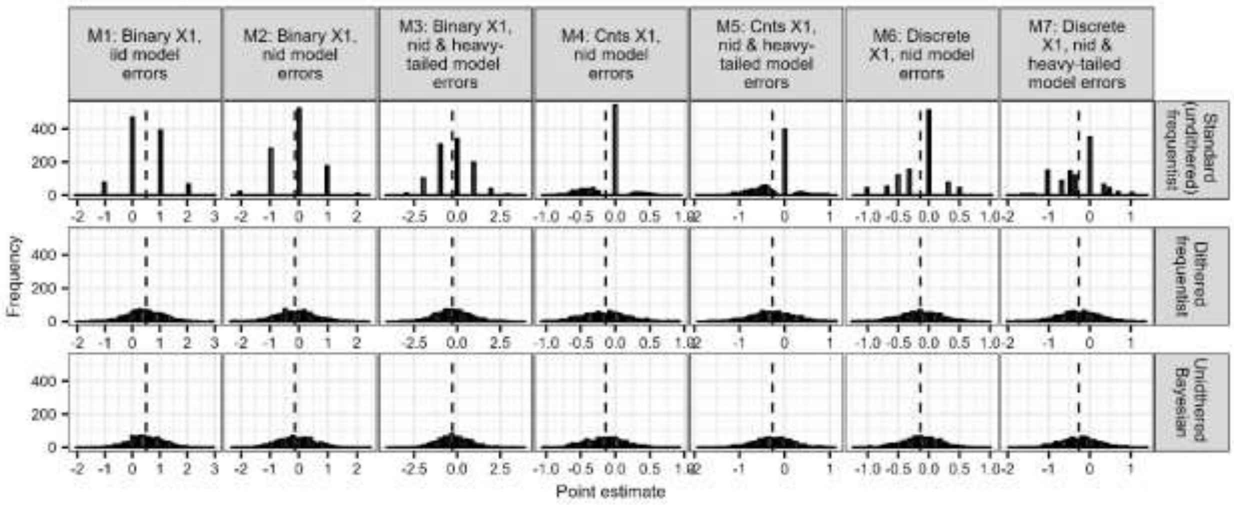

K) n = 750, τ = 0.5

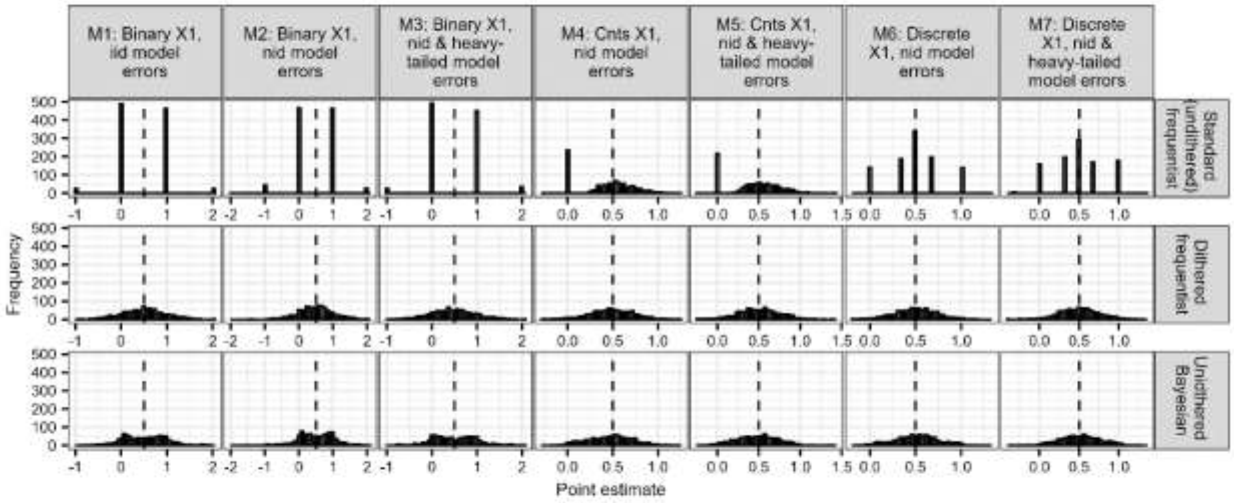

L) n = 750, τ = 0.9

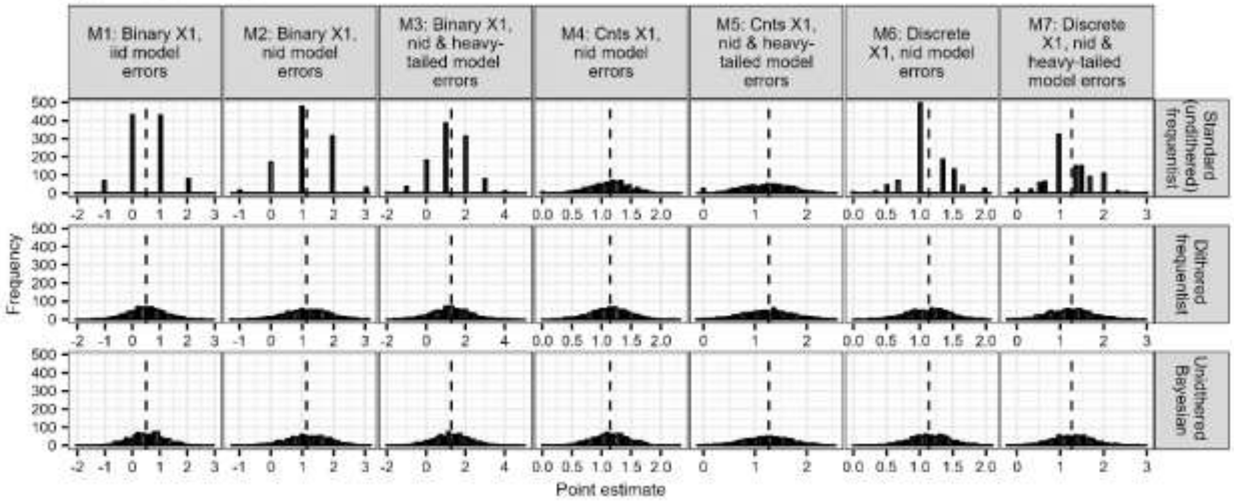

Figure S3. Trace plot for Bayesian quantile regression for all models and sample sizes considered. All trace plots have 10,000 draws with the first 2,000 draw removed (denoted by grey box). See https://github.com/jalam11/QR-with-discrete-outcomes for 9 additional trace plots from unique simulations.

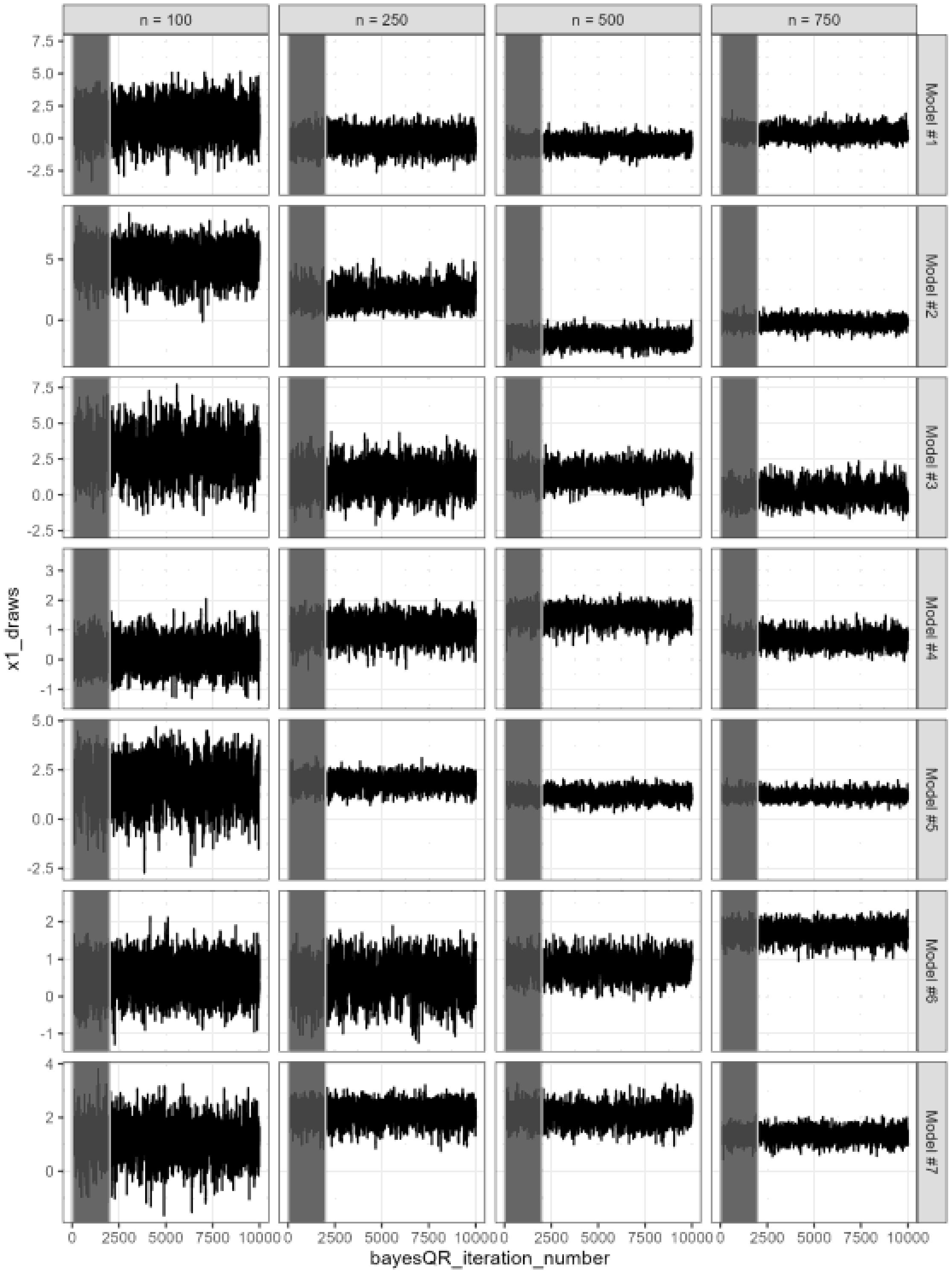

Figure S4. Comparison of the empirical coverage probabilities (circles) of quantile regression methods with a discrete outcome variable. Sample sizes of n=100 (A), n=500 (B), and n=750 (C) used (see manuscript for n = 250). The nominal value (horizontal dashed line) is 0.9. Arrows denote out of range values. Vertical lines depict Monte Carlo standard errors (often very small). Simulations with infinitely wide intervals (only observed with the undithered rank-based method) were excluded. Undithered xy method was not run on models 4 and 5 (continuous x_1) due to software crashes. In rare instances, Bayesian quantile regression failed to generate any valid results after 20 attempts.

Abbreviations: iid, independent and identically distributed errors; nid, non-independently distributed errors.

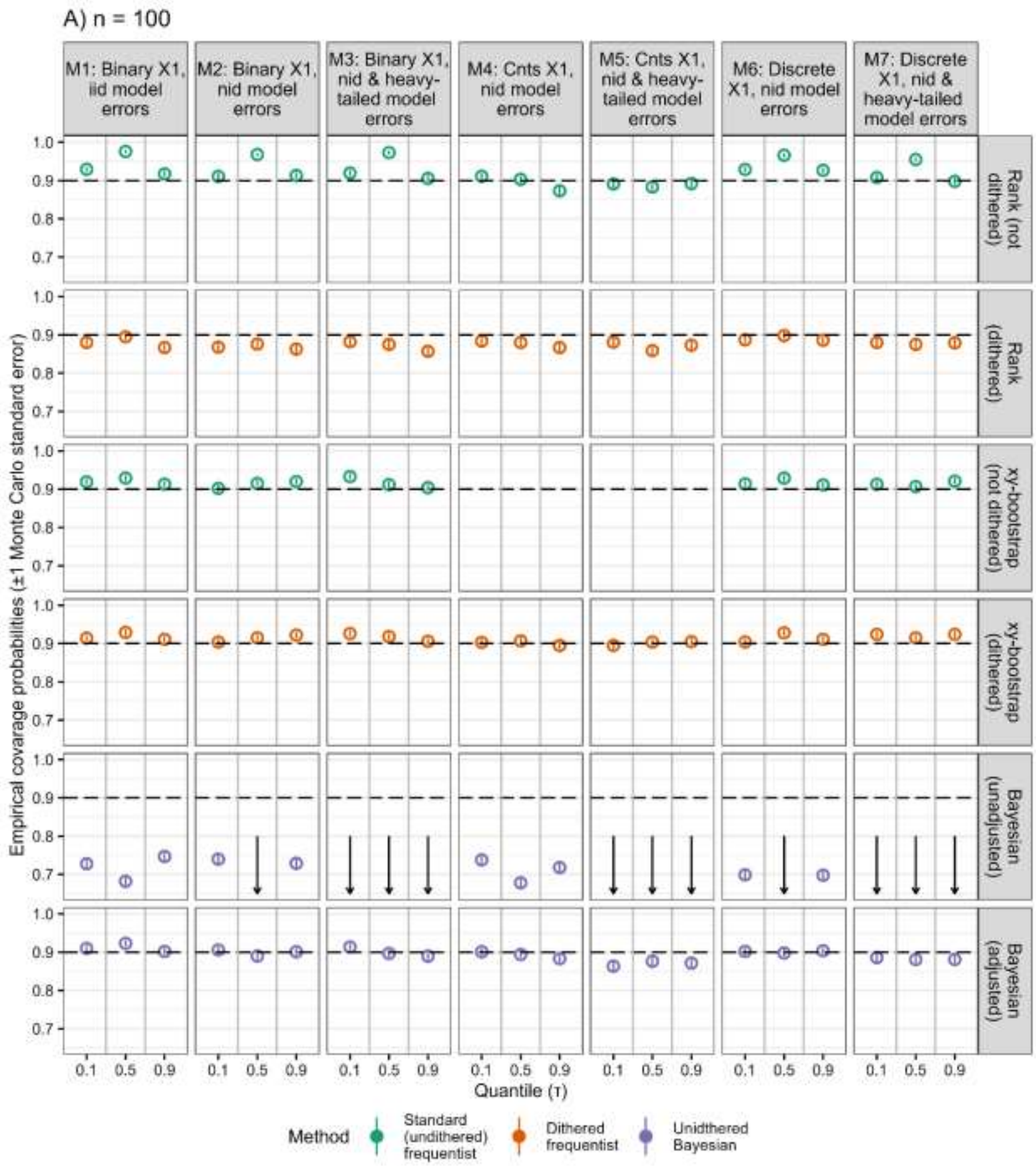

A) n = 100
M1: Binary X1, iid model errors
M2: Binary X1, nid model errors
M3: Binary X1, nid & heavy-tailed model errors
M4: Cnts X1, nid model errors
M5: Cnts X1, nid & heavy-tailed model errors
M6: Discrete X1, nid model errors
M7: Discrete X1, nid & heavy-tailed model errors
Rank (not dithered)
Rank (dithered)
xy-bootstrap (not dithered)
xy-bootstrap (dithered)
Bayesian (unadjusted)
Bayesian (adjusted)
Empirical covarage probabilities (±1 Monte Carlo standard error)
Quantile (τ)
Method
Standard (undithered) frequentist
Dithered frequentist
Unidthered Bayesian

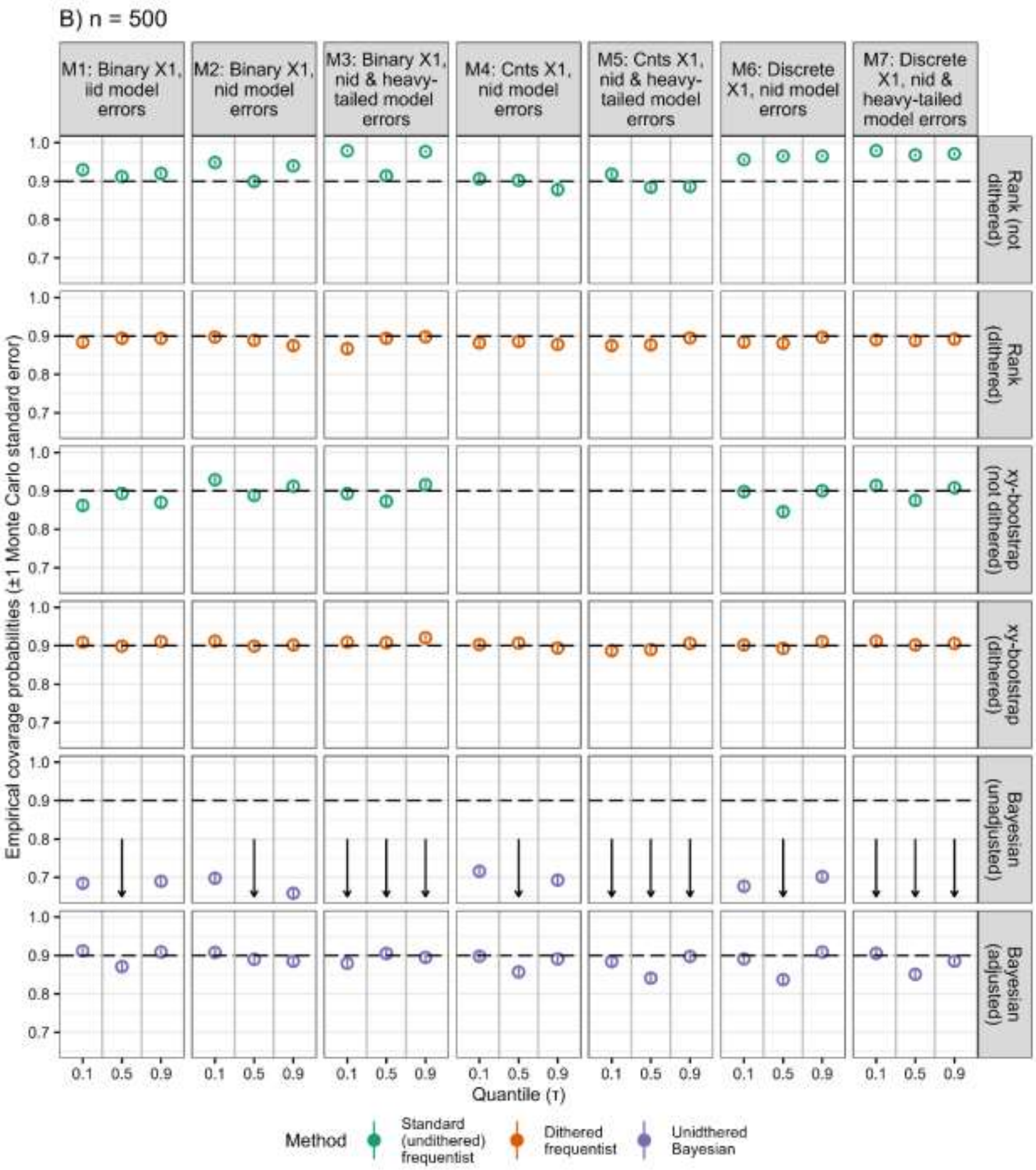
B) n = 500
M1: Binary X1, iid model errors
M2: Binary X1, nid model errors
M3: Binary X1, nid & heavy-tailed model errors
M4: Cnts X1, nid model errors
M5: Cnts X1, nid & heavy-tailed model errors
M6: Discrete X1, nid model errors
M7: Discrete X1, nid & heavy-tailed model errors
Rank (not dithered)
Rank (dithered)
xy-bootstrap (not dithered)
xy-bootstrap (dithered)
Bayesian (unadjusted)
Bayesian (adjusted)
Empirical covarage probabilities (±1 Monte Carlo standard error)
Quantile (τ)
Method
Standard (undithered) frequentist
Dithered frequentist
Unidthered Bayesian

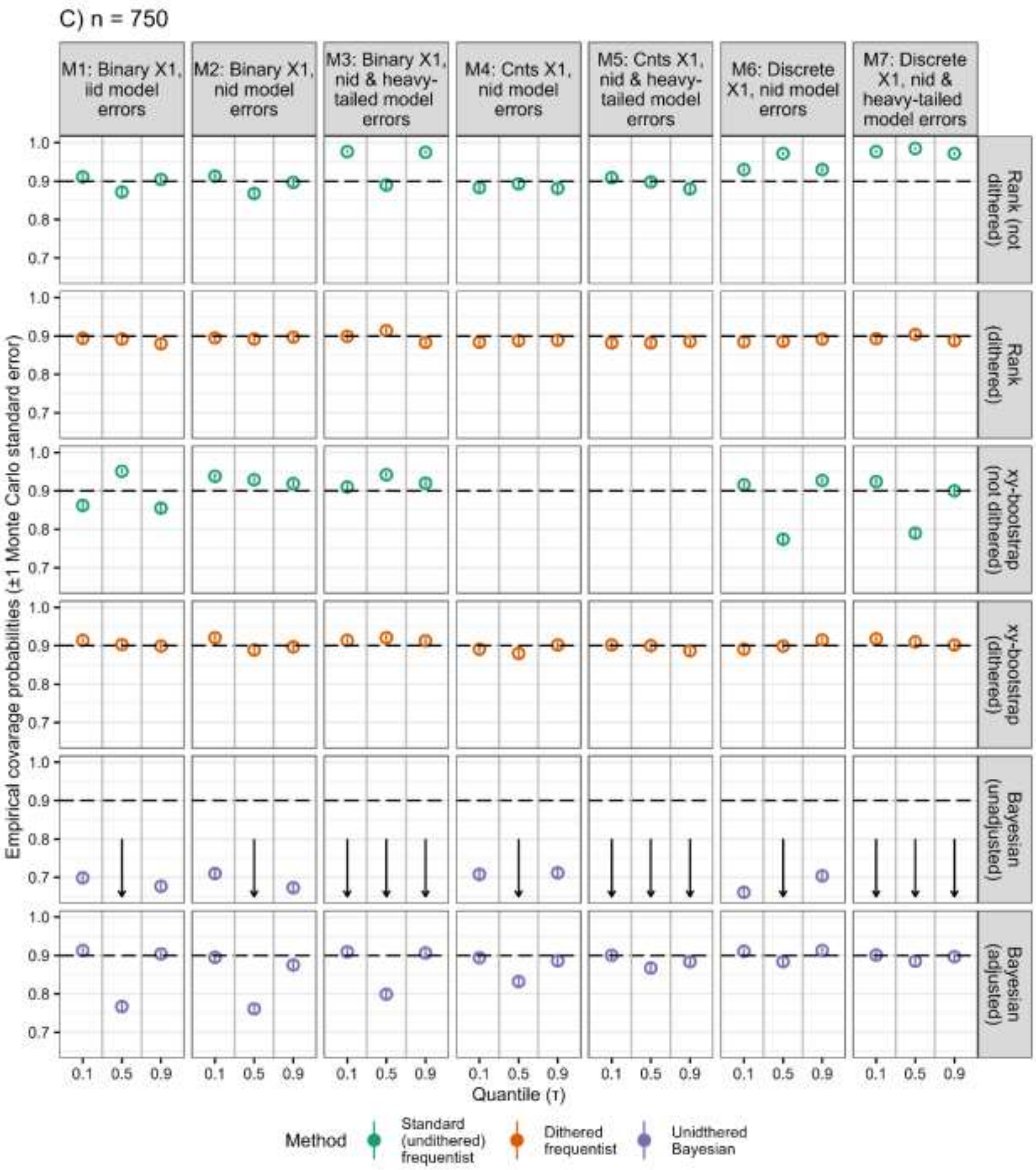
C) n = 750
M1: Binary X1, iid model errors
M2: Binary X1, nid model errors
M3: Binary X1, nid & heavy-tailed model errors
M4: Cnts X1, nid model errors
M5: Cnts X1, nid & heavy-tailed model errors
M6: Discrete X1, nid model errors
M7: Discrete X1, nid & heavy-tailed model errors
Rank (not dithered)
Rank (dithered)
xy-bootstrap (not dithered)
xy-bootstrap (dithered)
Bayesian (unadjusted)
Bayesian (adjusted)
Empirical covarage probabilities (±1 Monte Carlo standard error)
1.0
0.9
0.8
0.7
0.1
0.5
0.9
Quantile (τ)
Method
Standard (undithered) frequentist
Dithered frequentist
Undithered Bayesian

Figure S5. Comparison of the empirical standard deviation of the 90% intervals (circles) from quantile regression methods on a discrete outcome variable. The quantiles τ=0.1 (A) and 0.5 (B) were modelled (see manuscript for τ=0.9). Vertical lines depict Monte Carlo standard errors (often very small). Simulations with infinitely wide intervals (only observed with the undithered rank-based method) were excluded. Undithered xy method was not run on models 4 and 5 (continuous x_1) due to software crashes. In rare instances, Bayesian quantile regression failed to generate any valid results after 20 attempts.

Abbreviations: iid, independent and identically distributed errors; nid, non-independently distributed errors.

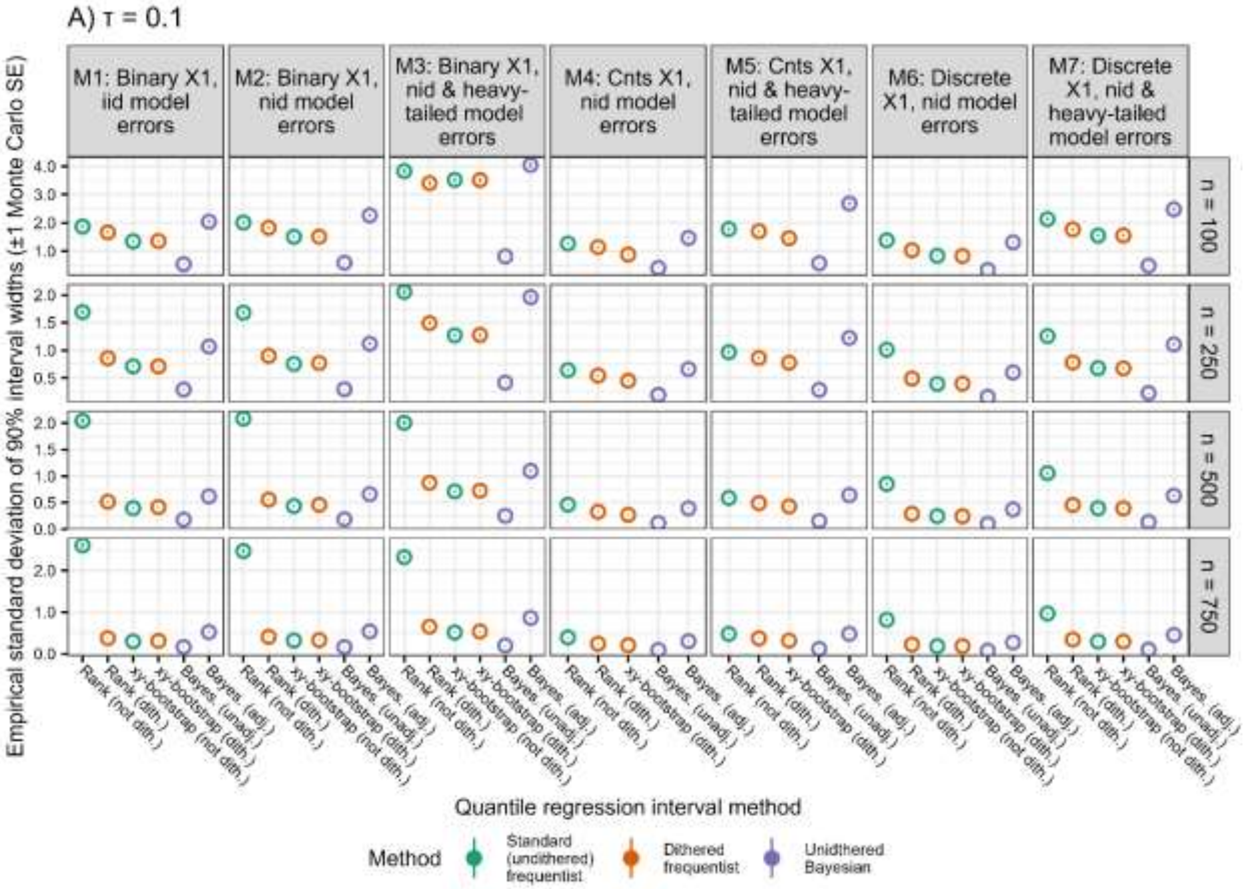

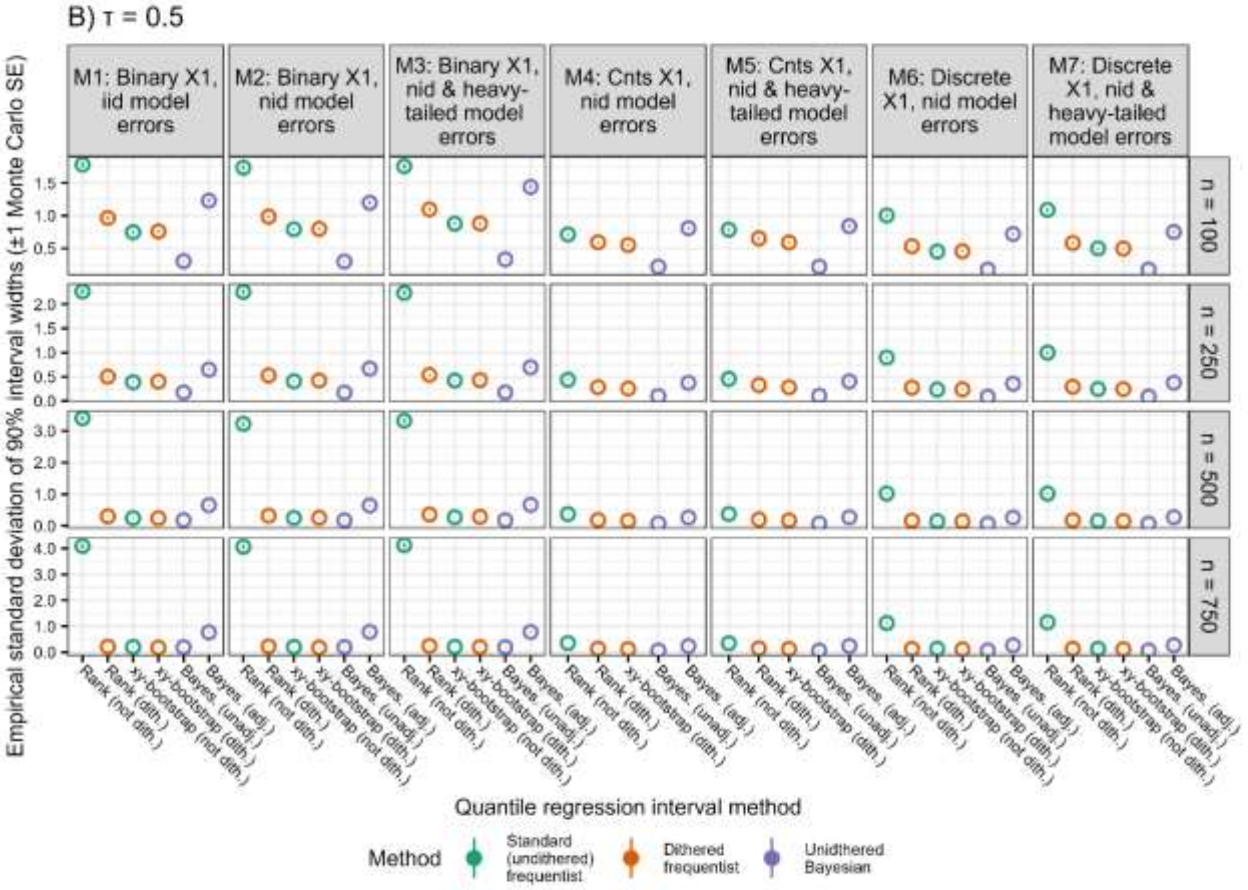
B) τ = 0.5
M1: Binary X1, iid model errors
M2: Binary X1, nid model errors
M3: Binary X1, nid & heavy-tailed model errors
M4: Cnts X1, nid model errors
M5: Cnts X1, nid & heavy-tailed model errors
M6: Discrete X1, nid model errors
M7: Discrete X1, nid & heavy-tailed model errors
n = 100
n = 250
n = 500
n = 750
Empirical standard deviation of 90% interval widths (±1 Monte Carlo SE)
Rank (not dith.)
Rank (dith.)
xy-bootstrap (not dith.)
xy-bootstrap (dith.)
Bayes. (unadj.)
Bayes. (adj.)
Quantile regression interval method
Method
Standard (undithered) frequentist
Dithered frequentist
Undithered Bayesian

References

1. Koenker R. Quantreg: quantile regression. *http://CRAN R-project org/package= quantreg*. Published online 2009. https://ci.nii.ac.jp/naid/10026704030/

2. Benoit DF, Van den Poel D. bayesQR: A Bayesian Approach to Quantile Regression. *J Stat Softw*. 2017;76(1):1-32.

3. Parzen MI, Wei LJ, Ying Z. A Resampling Method Based on Pivotal Estimating Functions. *Biometrika*. 1994;81(2):341-350.

4. Yang Y, Wang HJ, He X. Posterior Inference in Bayesian Quantile Regression with Asymmetric Laplace Likelihood. *Int Stat Rev*. 2016;84(3):327-344.

5. Koenker R. *Quantile Regression*. Cambridge University Press; 2005.
